# Synopsis of the *SOFL* Plant-Specific Gene Family

**DOI:** 10.1534/g3.118.200040

**Published:** 2018-02-23

**Authors:** Reuben Tayengwa, Jianfei Zhao, Courtney F. Pierce, Breanna E. Werner, Michael M. Neff

**Affiliations:** * Program in Molecular Plant Sciences and; † Department Crop and Soil Sciences, Washington State University, Pullman, Washington 99164-6420, and; ‡ Department of Biology, University of Pennsylvania, Philadelphia, Pennsylvania 19104

**Keywords:** SOFL, plant-specific, gene family, phylogenetic, *Arabidopsis*, evolution, introns

## Abstract

*SUPPRESSOR OF PHYB-4#5DOMINANT (sob5-D)* was previously identified as a suppressor of the *phyB-4* long-hypocotyl phenotype in *Arabidopsis thaliana*. Overexpression of *SOB5* conferred dwarf phenotypes similar to those observed in plants containing elevated levels of cytokinin (CK) nucleotides and nucleosides. Two SOB-FIVE- LIKE (SOFL) proteins, AtSOFL1 and AtSOFL2, which are more similar at the protein level to each other than they are to SOB5, conferred similar phenotypes to the *sob5-D* mutant when overexpressed. We used protein sequences of founding *SOFL* gene family members to perform database searches and identified a total of 289 *SOFL* homologs in genomes of 89 angiosperm species. Phylogenetic analysis results implied that the *SOFL* gene family emerged during the expansion of angiosperms and later evolved into four distinct clades. Among the newly identified gene family members are four previously unreported *Arabidopsis SOFLs*. Multiple sequence alignment of the 289 SOFL protein sequences revealed two highly conserved domains; SOFL-A and SOFL-B. We used overexpression and site-directed mutagenesis studies to demonstrate that SOFL domains are necessary for *SOB5* and *AtSOFL1’s* overexpression phenotypes. Examination of the subcellular localization patterns of founding *Arabidopsis thaliana* SOFLs suggested they may be localized in the cytoplasm and/or the nucleus. Overall, we report that *SOFLs* are a plant-specific gene family characterized by two conserved domains that are important for function.

Many genes can be grouped into specific families based on nucleotide and protein sequence similarity. Such gene families have arisen as a result of duplication and expansion of individual members (Wang *et al.* 2012). Gene family sizes vary across different lineages and may have important functional outcomes related to adaptation or speciation (Qu and Zhu 2006; Vollrath *et al.* 1998). Functional and evolutionary studies of genes belonging to various families have greatly enhanced our understanding of components involved in plant growth and development (Kim *et al.* 2007; Higuchi *et al.* 2004; Xie *et al.* 1999; Kim 2006; Kondou *et al.* 2010; Zhao *et al.* 2014). Despite significant progress made toward discovery and curations of many gene families, the majority of plant gene families remain uncharacterized (Guo 2012). Therefore, the continued identification and study of hitherto uncharacterized gene families remains crucial for our continued endeavor to eventually achieve the goal of functionally characterizing most of the genes.

The *Arabidopsis thaliana* activation-tagged mutant *sob5-D (SUPPRESSOR OF PHYB-4#5DOMINANT)* was previously identified as a suppressor of the long-hypocotyl phenotype conferred by a *phyB-4* mutation (Zhang *et al.* 2006). Database searches using SOB5 (At5g08150) protein sequence as a query revealed two SOB-FIVE- LIKE (SOFL) proteins in *Arabidopsis*, AtSOFL1 (At1g26210) and AtSOFL2 (At1g68870) (Zhang *et al.* 2006). AtSOFL1 and AtSOFL2 are more similar to each other than they are to SOB5 at the protein level (Zhang *et al.* 2006, 2009). When individually overexpressed, *AtSOFL1* and *AtSOFL2* conferred phenotypes similar to the *sob5-D* activation-tagged mutant (Zhang *et al.* 2006, 2009). Transgenic lines individually overexpressing *SOB5*, *AtSOFL1* and *AtSOFL2* genes displayed reduced apical dominance, conferred smaller rosette leaves, retarded root growth and delayed senescence phenotypes (Zhang *et al.* 2006, 2009). In addition, these transgenic lines also contained elevated levels of cytokinin (CK) nucleotides and nucleosides; *trans*-zeatin riboside (tZR), *trans*-zeatin riboside monophosphate (tZRMP), and *N^6^*-(∆^2^-isopentenyl)adenosine monophosphate (iPRMP) (Zhang *et al.* 2006, 2009). These results suggested that the founding *SOFL* gene family members may have similar or overlapping CK-related functions. However, the specific details of what roles, if any, intermediate CK nucleotide/nucleoside species play during plant growth and development are not fully understood and will have to be further investigated in the future.

Zhang *et al.*, (2006), reported that the three founding *SOFLs* constituted a small, novel and intron-less *Arabidopsis thaliana* gene family. In addition, *SOFL* homologs and expressed sequence tags were also identified in a few monocotyledonous and dicotyledonous species; *Malus x domestica*, *Brassica napus*, *Oryza sativa* and *Populus trichocarpa* (Zhang *et al.* 2006). Due to few sequenced genomes at the time it was difficult to obtain a comprehensive phylogenetic overview of the *SOFL* gene family. Nonetheless, based on these preliminary results we speculated that *SOFL* homologs also existed in genomes of other eukaryotic and/or prokaryotic species (Zhang *et al.* 2006).

Since *Arabidopsis SOFLs* were first identified, more genome sequence information has been released (Fang *et al.* 2013; Wheeler *et al.* 2008; Kersey *et al.* 2016). We have taken a phylogenetic approach to update our understanding of the evolution and diversification of the *SOFL* gene family. Database searches using the three founding SOFL members’ proteins sequences as queries revealed at least 289 *SOFL* homologs in 89 angiosperm species. During this process, we also discovered that the *Arabidopsis thaliana* genome contains four additional *SOFLs* that were previously unreported. In addition, phylogenetic analysis showed that the 289 *SOFL* homologs evolved into four main clades. Finally, to gain further insights into this gene family, we performed subcellular localization, tissue expression and domain mutational studies in three founding *SOFL* members. This updated synopsis of the *SOFL* gene family provides basic background information needed to design future studies to eventually fully characterize this novel gene family.

## Materials AND METHODS

### Plant materials and growth conditions

All *Arabidopsis thaliana* lines used in this manuscript are in the *Columbia* (Col-0) background. For phenotypic analysis, all plants were grown on soil in pots in growth chambers set at 21° under white light (200 μmol m-^2^ sec-^1^) and 60–70% humidity, and under 16 hr light and 8 hr darkness.

### Sequence alignment and phylogenetic analysis

SOB5, AtSOFL1 and AtSOFL2 protein sequences were downloaded from NCBI (Wheeler *et al.* 2008; Sayers *et al.* 2009). The sequences were used as queries to search NCBI and Phytozome (Goodstein *et al*. 2011) databases for *SOFL* homologs using BLASTP option using a < 1 X 10-2 cut-off value. The amino acid sequences were aligned using Probalign V1.3 (Roshan 2014) on CIPRES Science Gateway Server (Miller *et al.* 2015). Bayesian inference analysis was performed with the Mr. Bayes 3.2.1 on XSEDE tool on CIPRES Science Gateway for 30 million generations with 8 chains to run. Generations were sampled every 10,000 generations with the first 25% generations used as a burn-in.

### Sequence logo analysis

Sequence logo analysis of conserved SOFL-A and SOFL-B domains was performed using the online WebLogo tool (Crooks *et al.* 2004). The 289 SOFL sequences retrieved from NCBI (Sayers *et al.* 2009) and Phytozome (Goodstein *et al.* 2011) were used to generate the sequence logo using default settings.

### Overexpression and point-mutation analysis of SOFL domains in SOB5 and AtSOFL1

Site-directed point mutations were generated using the QuikChange® Lightning mutagenesis kit according to manufacturer’s instructions (Agilent Technologies). Mutagenesis primers were designed using Agilent’s web-based QuikChange® Primer Design Program (http://www.genomics.agilent.com/primerDesignProgram.jsp) (Table S1). Gateway® compatible entry vectors, pENTR223 (ABRC), carrying *SOB5* and *AtSOFL1* coding sequences, were used as templates in mutagenesis PCR reactions. Each point mutation was confirmed via sequencing. Once mutagenesis was confirmed, *SOB5* or *AtSOFL1* coding sequences carrying point mutations were cloned via LR® reactions into pCHF3 (Zhang *et al.* 2006) and pEarlyGate100 (Earley *et al.* 2006) binary vectors, respectively. Destination binary vectors carrying mutated *SOB5* and *AtSOFL1* genes under control of the constitutive 35S promoter were transformed into *Arabidopsis thaliana* Col-0 wild-type plants using the floral dip method and the *Agrobacterium tumafaciens* strain GV3101 (Clough and Bent 1998). Transformants were screened on 1/2× Linsmaier and Skoog media plates containing 25 mg L^-1^ Basta (pEarleyGate100) and 30 mg L^-1^ kanamycin (pCHF3). Homozygous lines were identified in the T_3_ generation. Three independent transgenic lines representing each construct were selected for analysis. All plants were grown and analyzed in a growth chamber.

### RNA extraction and real-time PCR

Total RNA was extracted from tissues pooled from three independent plants (n = 3 for all tissues) of six-day old seedlings, 10-day old juvenile plants, rosette leaves from a 20-day old plant, floral tissue, siliques, a 25-day old adult plant, and roots from a 25-day old plant using the Qiagen RNeasy® Mini Kit (Qiagen). On-column DNase digestion was performed using the RNase-Free DNase Set (Qiagen, Valencia, CA). Complementary DNA (cDNA) was further generated using the iScript® Reverse Transcription Supermix for RT-qPCR (Bio Rad, Hercules, CA). Identical amounts of cDNA input were used in PCR reactions using either *ubiquitin10 (UBQ10)* or gene-specific primers (Table S1). Amplification using *UBQ10* primers was done in 30 cycles while 45 cycles were used for gene-specific primers. Negative control, no reverse transcriptase (No RT), samples were prepared using the same reaction conditions and reagents (minus reverse transcriptase enzyme) used to make cDNA.

### CFP-SOFL fusion constructs and onion bombardment

To generate pSAT4-CFP-SOB5, pSAT4-CFP-AtSOFL1, and pSAT4-CFP-AtSOFL2 N-terminal fusion constructs, respective full-length coding sequences, each contained in the entry vector pENTR223 (ABRC), were cloned into pSAT4-CFP (ABRC) destination vector via Gateway® LR reactions (Invitrogen, Carlsbad, CA). Onion epidermal cells were co-bombarded with pSAT6-mRFP (ABRC) plasmid and pSAT4-CFP constructs carrying *SOFLs* fused to CFP using a PDS-1000/He Biolistic transformation system (Bio Rad, Hercules, CA). Bombarded onion epidermal layers were incubated in the dark for 40 hr. To identify successfully transformed cells and observe fluorescent signals, the onion epidermal layer was examined on a Leica TCS SP8 X (Leica Microsystems, Mannheim, Germany) confocal microscope.

### Data availability

Genetic material used in this manuscript is available upon request. Primers used in the study are listed in Table S1. Total numbers of *SOFL* homologs identified in various species are listed in Table S2. Table S3 shows results from protein localization prediction analysis. Alternatively-spliced *AtSOFL1* product sequences (*AtSOFL1.1* and *AtSOFL1.2*) are provided in File S2. Figure S1 shows a cartoon and gel image of *AtSOFL1*’s alternative splicing products. Sequences extracted from database searches using the founding SOFL protein sequences as queries are provided in File S1.

## Results

### SOFLs are a plant-specific gene family

**Figure 1:**
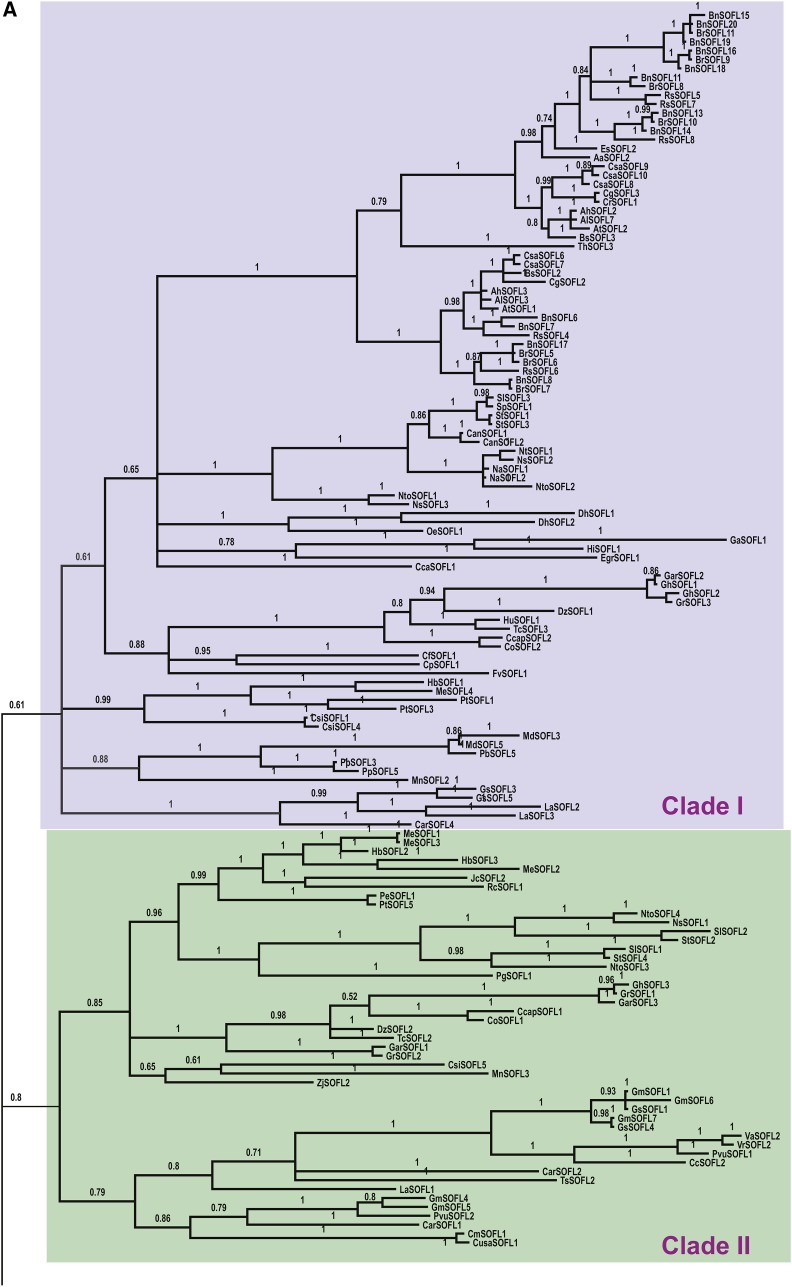

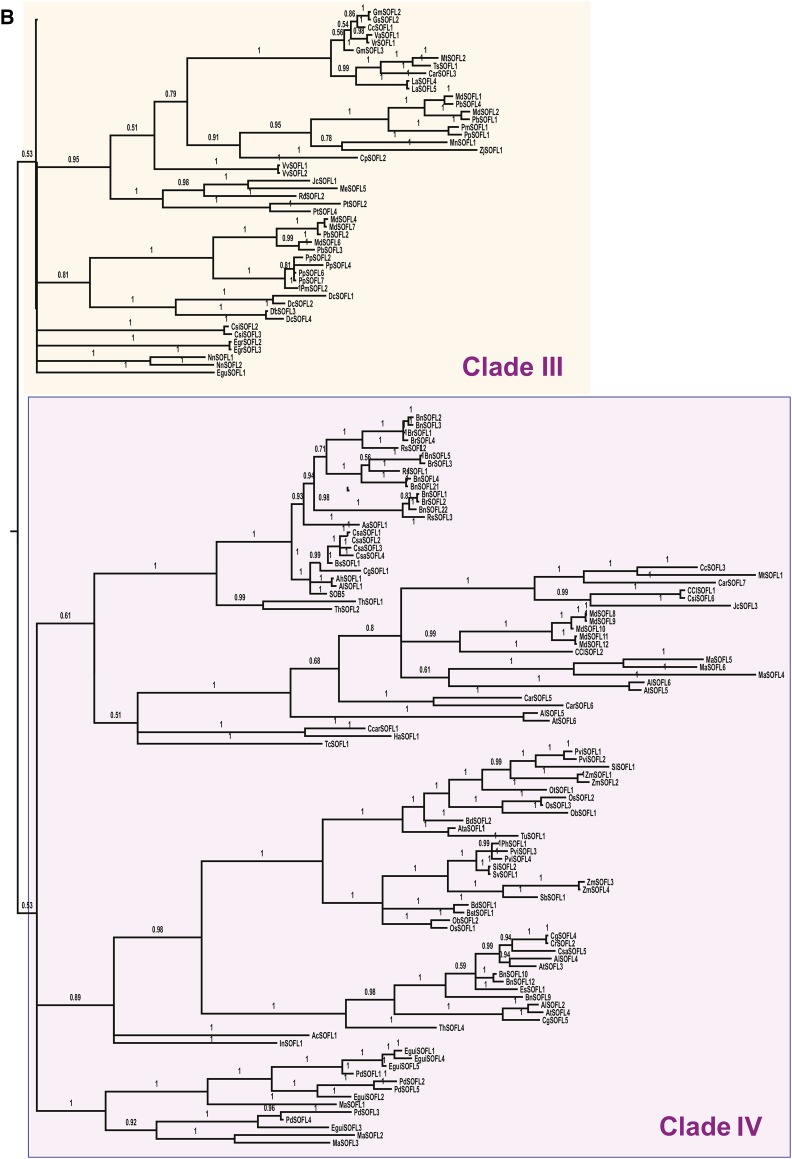
Phylogenetic analysis of 289 SOFL homolog protein sequences showed that the gene family evolved into four main clades. (A) and (B) Annotated rectangular layout mid-point root Mr. Bayes phylogenetic trees. Bayesian inference analysis was performed on 289 SOFL homologs amino acid sequences with the Mr. Bayes 3.2.1 on XSEDE tool on CIPRES Science Gateway for 30 million generations with 8 chains to run with convergence at 0.0099 (Miller *et al.* 2015).

To identify SOFL homologs, we performed BLASTP searches against the NCBI database (Sayers *et al.* 2009) and Phytozome database (Goodstein *et al*. 2011) using SOB5, AtSOFL1 and AtSOFL2 sequences as queries. We extracted 289 SOFL homologs from 89 flowering plant species, of which 15 were monocotyledons and 74 were dicotyledons (Table S2). To gain insight into the evolutionary history of the *SOFL* gene family, we next performed phylogenetic analysis using the 289 SOFL protein sequences extracted via database searches. Our analysis suggested that *SOFLs* likely evolved into four major clades: Clade I, II, III and IV (Figure 1a and 1b). We hypothesize that *SOFL*s within each clade have greatly expanded through evolution, with Clades I and -IV showing the largest expansion (Figure 1a and 1b). However, since the tree was constructed only with the existing *SOFL* genes in the plant species examined and no outgroup used, we cannot rule out gene loss events. Annotation data from NCBI (Sayers *et al.* 2009), Phytozome (Goodstein *et al.* 2011) and individual species genome databases revealed that 60 *SOFL* homologs contained introns and another 226 did not (Table S2). There was no annotation data for *Arabis alpina* and *Erythranthe guttata (Mimulas guttatus) SOFL* homologs.

### Arabidopsis thaliana genome contains seven SOFL members

It was previously reported that the *Arabidopsis* genome contained three *SOFL* members (Zhang *et al.* 2006, 2009). However, latest database search results revealed that the *Arabidopsis thaliana* genome contained a total of seven *SOFL* members (File S1). In addition, we also identified seven *SOFLs* in *Arabidopsis lyrata* (File S1). We have designated the newly identified *Arabidopsis thaliana SOFL* genes as *AtSOFL3* (AT3G30580), *AtSOFL4* (AT5G38790), *AtSOFL5* (AT4G33800) and *AtSOFL6* (AT1G58460). *SOB5* is in Clade IV of the phylogenetic tree (Figure 1a and 1b), compared to *AtSOFL1* and *AtSOFL2* which were in Clade I. Interestingly, the newly identified *SOFL* members, *AtSOFL3*, *AtSOFL4*, *AtSOFL5* and *AtSOFL6* are in Clade IV (Figure 1b), suggesting that genetic redundancy may exist among these genes and *SOB5*. All *Arabidopsis SOFLs* are intron-less except *AtSOFL1* (recently classified by TAIR as intron-containing), *AtSOFL5* and *AtSOFL6*.

**Figure 2:**
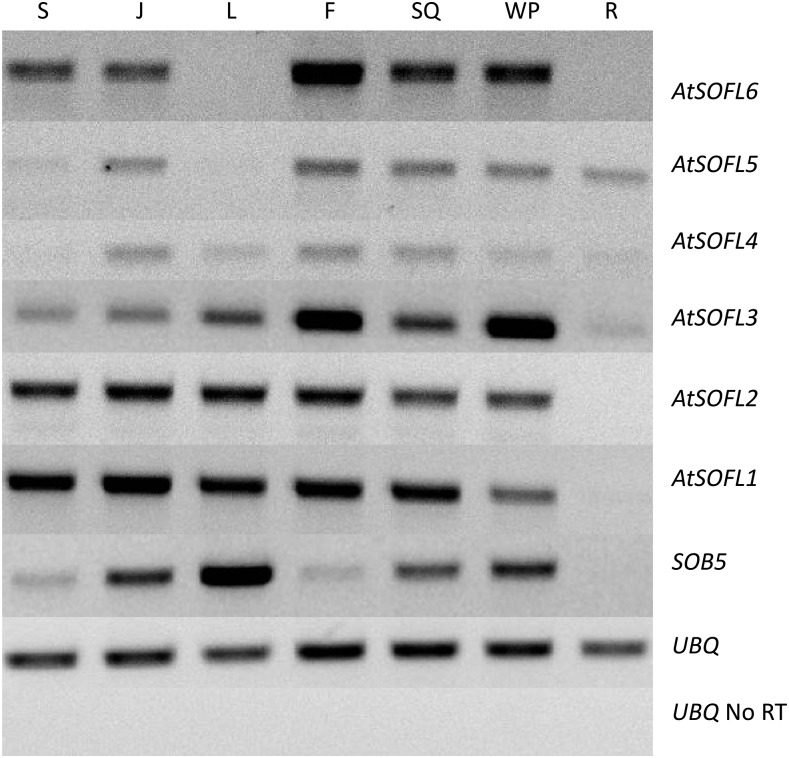
Expression levels of *Arabidopsis thaliana SOFL* genes in various tissues. Total RNA was isolated from; 6-day old seedlings (S), juvenile plants (J), rosette leaf (L), floral structure (F), siliques (SQ), whole adult plant (WP) and roots (R) and used for RT-PCR analysis. *UBIQUITIN10* was used as an internal control for PCR. No reverse transcriptase (No RT) RNA samples were used as negative controls.

We next assessed the expression patterns of *Arabidopsis thaliana SOFL* homologs. RNA was extracted from seedlings, juvenile plants, adult rosette leaf, floral structures, siliques, an entire flowering plant and adult plant roots. *SOB5*, *AtSOFL1* and *AtSOFL2* transcripts were detected in all samples tested except roots (Figure 2). *AtSOFL3*, *AtSOFL4*, *AtSOFL5* and *AtSOFL6* were expressed at varying levels in seedlings, juvenile plants, flowers, siliques and whole plant (Figure 2). *AtSOFL3*, *AtSOFL4*, *AtSOFL5* were the only ones detected in roots. *AtSOFL6* showed little to no expression in rosette leaves and roots, and similarly, *AtSOFL5* showed little to no expression in seedlings and rosette leaves (Figure 2). *AtSOFL4* was expressed in all samples tested but had the lowest transcript levels overall. The varying transcript accumulation levels and differential tissue expression patterns may suggest unique functional roles among the seven *Arabidopsis thaliana SOFLs* during plant development (Figure 2). Surprisingly, during efforts to amplify a full-length *AtSOFL1* (At1g26210) cDNA we identified a previously unreported splice variant. We have designated the original 447 bp transcript *AtSOFL1* (At1g26210.1), and the newly identified 771bp second transcript as (At1g26210.2) (Figure S1a, b).

### SOFL homologs are characterized by two conserved domains

**Figure 3:**
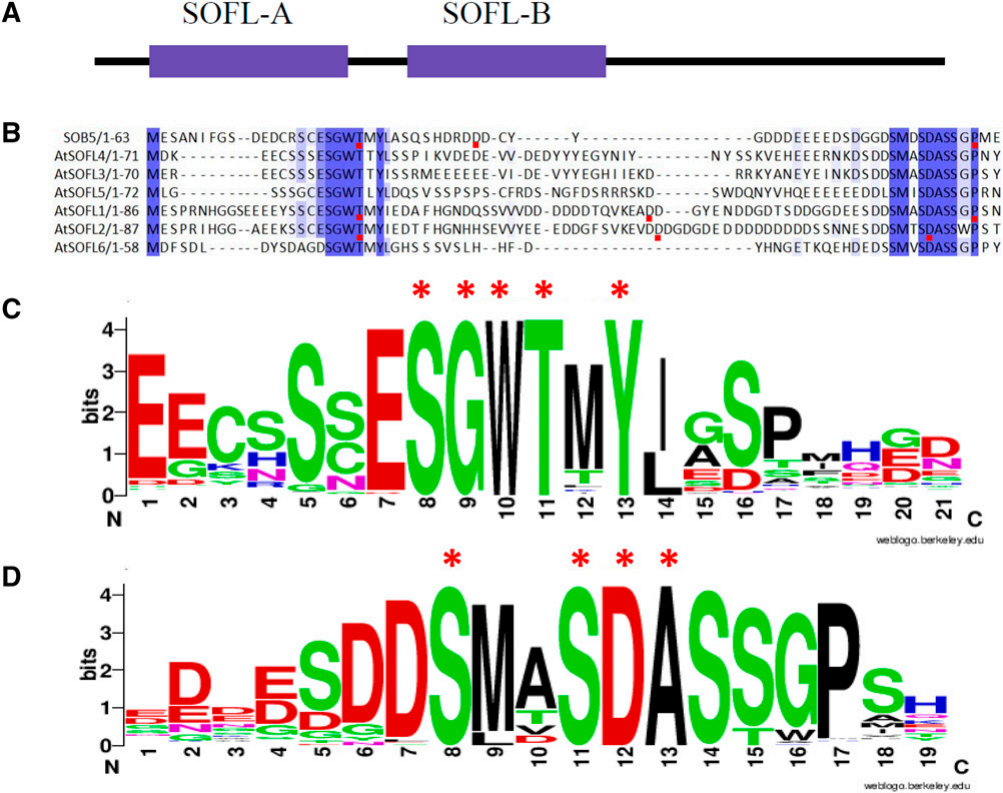
Multiple sequence alignment and analysis of SOFL proteins. (a) Illustration showing the topology of SOFL proteins. Purple rectangular blocks represent the two conserved domains, SOFL-A and SOFL-B. (b) Partial N-terminal amino acid sequence alignment of *Arabidopsis thaliana* SOFL proteins. The alignment was obtained using JalView Probcons with Defaults program (Malek 2001; Zhang 2003). Semi-conserved amino acids are indicated with a light purple shade. Conserved amino acids are indicated by a darker purple shade. Red squares indicate amino acid residues that were selected for site-directed mutagenesis. WebLogo© analysis of conserved, (c) SOFL-A domain, and (d) SOFL-B domain, using all 289 SOFL protein sequences (Lin and Hu 2013). Red asterisk denotes 100% conserved amino acid residues among all 289 SOFL sequences.

Multiple sequence alignment analysis is a useful tool used to infer relationships among gene family members. Alignment data can provide functional information through the identification of key conserved domains and other important features. We aligned SOFL homologs protein sequences and identified two conserved domains in the N-terminal region (Figure 3a), which were previously reported by Zhang *et al.* (2006), albeit based on fewer protein sequences. We have designated the two conserved domains, SOFL-A and SOFL-B (Figure 3a). When only *Arabidopsis* SOFL sequences were aligned (Figure 3b) and compared to an alignment of all 289 SOFL homologs (Figure 3c, 3d) we observed slight differences in the SOFL-B domain. In an alignment of *Arabidopsis* SOFL sequences only, SOFL-B domain contains eight 100% conserved residues SM×SDASS×P (Figure 3b). However, the same domain only contains four 100% conserved residues (S××SDA) when all 289 SOFLs homologous sequences were aligned (Figure 3d). In general, based on all SOFL ortholog sequences identified so far, SOFL-A domain contains an SGWT×Y motif and SOFL-B domain contains an S××SDA motif (× = amino acid residue that is not 100% conserved) (Figure 3c, d). There were no areas of high sequence similarity in the C-terminal region, except for an increased presence of basic amino acid residues in most SOFL homologs (Table S3).

### Conserved amino acid residues in SOFL domains are required for the manifestation of SOB5 and AtSOFL1 overexpression phenotypes

Previously, Zhang *et al.*, (2009), investigated the requirement of some of the 100% conserved residues in SOFL-A and SOFL-B domains for the manifestation of *AtSOFL2’s* overexpression phenotype via site-directed mutagenesis. Constructs carrying *AtSOFL2* gene harboring individual point mutations in each of the two conserved domains were used to generate plants overexpressing an aberrant protein. Resultant transgenic mutant plants overexpressing *AtSOFL2* gene harboring T21I and D80N point mutations lost the phenotype that is typically associated with the overexpression of the wild type *AtSOFL2* gene (Zhang *et al.* 2009). These results implied that the conserved amino acid residues were important for the manifestation of *AtSOFL2* overexpression phenotype.

**Figure 4:**
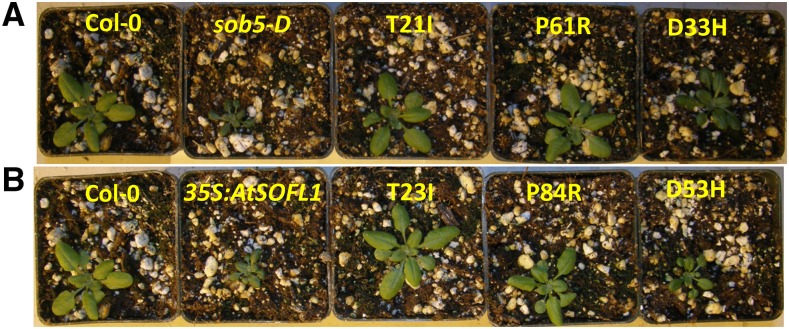
Phenotypes of transgenic plants overexpressing full-length *SOB5* and *AtSOFL1* genes harboring point mutations generated via site-directed mutagenesis. (a) Wild-type and *sob5-D* control plants compared to transgenic plants overexpressing the following *SOB5* point mutations: T21I, P61R and D33H. D33H mutation is in a non-conserved portion of *SOB5* gene. (b) Wild-type and *35S:AtSOFL1* control plants compared to transgenic plants overexpressing the following *AtSOFL1* point mutations: T23I, P84R and D53H. D53H is in a non-conserved portion of *AtSOFL1* gene. All plants were grown together in a growth chamber for two weeks. Three independent transgenic lines were analyzed. This experiment was repeated three times with similar outcomes.

To further investigate the biological importance of some of the 100% conserved residues in the other two founding SOFLs, SOB5 and AtSOFL1, we generated a series of site-directed point mutations in their respective SOFL-A and SOFL-B domains. Wild-type *Arabidopsis thaliana* plants were separately transformed with constructs carrying mutations in *SOB5* (T21I and P61R) and *AtSOFL1* (T23I and P84R) coding sequences. Transgenic plants overexpressing *SOB5* (T21I, P61R) and *AtSOFL1* (T23I, P84R) mutated genes lost the dwarf/semi-dwarf phenotypes typically observed when wild-type versions were overexpressed (Figure 4a, b). We also performed site-directed mutagenesis on non-conserved amino acid residues; D33H and D53H for SOB5 and AtSOFL1, respectively, which are not located in the two conserved domains (Figure 3b). Transgenic plants overexpressing mutated *SOB5* (D33H) and *AtSOFL1* (D53H) exhibited phenotypes similar to those observed when wild-type genes are constitutively expressed (Figure 4a, b) (Zhang *et al.* 2006, 2009). These data suggest that less conserved amino acid residues are not required for the overexpression phenotypes.

### SOFL subcellular localization

We used two publicly available online prediction programs, Wolf PSORT (Horton *et al.* 2007) and SeqNLS (Lin and Hu 2013), to gain insights into subcellular localization patterns of the 289 *SOFL* homologs. Wolf PSORT converts protein amino acid sequences into numerical localization features based on sorting signals, amino acid composition and functional domains such as DNA-binding motifs (Horton *et al.* 2007). SeqNLS uses a sequential pattern mining algorithm to effectively identify potential nuclear localization signals (NLS) in protein sequences (Lin and Hu 2013). Wolf PSORT predicted 274 SOFL homologs to localize to the nucleus, with the remainder predicted to localize to chloroplast, cytoplasm, mitochondria, peroxisome, Golgi and extracellular space (Table S3). In contrast, the SeqNLS program detected nuclear localization signals in only 156 SOFLs with a statistically significant prediction score of at least 0.89 (Table S2) (Lin and Hu 2013). Interestingly, SeqNLS did not detect nuclear localization signals in 13 SOFLs which were predicted to localize to the nucleus by Wolf PSORT (Table S3). 119 SOFLs scored below the default cutoff threshold to be classified as containing a NLS. Overall, at least 156 SOFL homologs were predicted to localize to the nucleus by both Wolf PSORT and SeqNLS algorithms (Table S3).

**Figure 5:**
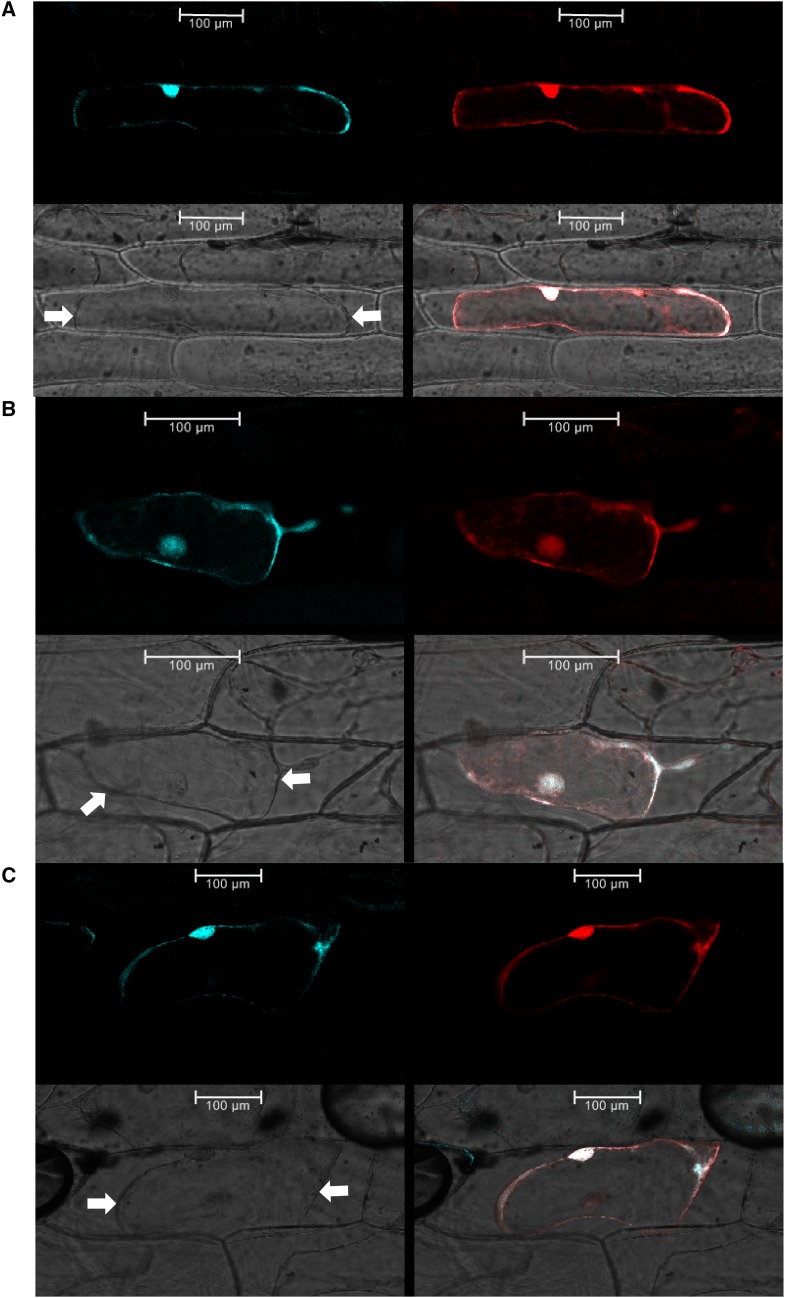
Subcellular localization of CFP-SOB5 (a), CFP-AtSOFL1 (b), CFP-AtSOFL2 (c) fusion proteins in onion epidermal cells. Most biochemical functions carried out in plant cells are performed by proteins in specific cellular locations. Protein subcellular localization studies, via fluorescent protein fusions, are a useful tool to narrow down cellular functions of novel or unknown proteins. Onion epidermal peels were biolistically bombarded with CFP-protein fusion constructs and incubated in the dark for 40 hr, then visualized under a confocal microscope. Each panel shows, in a clockwise direction, CFP-protein fusion signal, free RFP signal, merge of CFP and RFP signal and bright-field view showing outline of cells. White arrows indicate outline of plasmolyzed membrane. Free RFP construct was used as a control for successful bombardment as well as a localization marker. Plasmolysis was induced by treatment with 0.8 M mannitol.

To further assess some of the subcellular prediction data, we examined the intracellular distribution of the founding *Arabidopsis thaliana* SOFL members. We fused SOB5, AtSOFL1 and AtSOFL2 to the carboxyl end of a cyan fluorescent protein (CFP) and transiently expressed the fusion proteins in onion epidermal cells. CFP-SOB5, CFP-AtSOFL1 and CFP-AtSOFL2 fluorescent signals were observed in both the cytoplasm and nucleus of the onion epidermal cells (Figure 5). We also examined whether the fluorescent signals of all three fusion proteins were localized to the cytosol and not the cell wall. This was achieved by inducing plasmolysis through exposure of onion epidermal peels to 0.8 M mannitol. Ensuing the mannitol treatment, CFP signal remained restricted to the edges of the plasma membrane and/or cytoplasm, suggesting that CFP-SOB5, CFP-AtSOFL1 and CFP-AtSOFL2 fusion proteins were not localized to the cell wall.

## Discussion

### SOFL homologs emerged in angiosperms

The founding members of the *SOFL* gene family were first identified via an activation tagging screen in *Arabidopsis thaliana* (Zhang *et al.* 2006). Based on the limited number of sequenced genomes available at the time, it was initially concluded that *SOB5*, *AtSOFL1* and *AtSOFL2* were a small three-member intron-less *Arabidopsis thaliana* gene family. To expand on earlier studies and acquire a basic level understanding of this poorly characterized gene family, we took advantage of an increasing number of sequenced genomes to perform database searches using the founding *Arabidopsis thaliana* founding SOFL members as queries. We have retrieved a total of 289 SOFL sequences from 89 angiosperm species (Table S2). However, we cannot rule out the possibility that *SOFL* homologs are present in unsequenced organisms or were lost in other genomes. In addition, we expect additional *SOFL* homologs to be identified in other species as more genomes are sequenced. Furthermore, as genome sequence annotation improves, it is possible that some of the SOFL homologs currently classified as intron-less or intron-containing may be re-categorized in the future.

No *SOFL* homologs were identified in *Volvox cartari* (green algae), *Chlamydomonas reinhardtii* (green algae), *Ostreococcus lucimarinas* (single-celled water algae), *Micromonas pusilla* (water algae), *Physcomitrella patens* (non-vascular bryophyte, moss) and *Selaginella moellendorffii* (member of an ancient vascular plant lineage) (Lamesch *et al.* 2012), strongly suggesting that *SOFLs* may have emerged after the separation of lycophytes, pterophytes and seed plants. Out of the four main plant groups: bryophytes, seedless vascular (pteridophytes/lycophytes), gymnosperms and angiosperms, *SOFLs* have so far only been identified in angiosperms. We hypothesize that the *SOFL* gene family emerged and expanded during the evolution of flowering plant species.

### SOFL gene family is comprised of intron-containing and intron-less genes

Approximately 20% of *SOFL* homologs identified so far contain introns and 80% are intron-less (Table S2). These results are inconsistent with overall data from rice and *Arabidopsis* genomes which revealed the presence of only 19.9% and 21.7% intron-lacking genes, respectively (Jain *et al.* 2008). Our latest database search results also showed that *Arabidopsis thaliana* contains seven *SOFL* genes, in contrast to previous reports of three intron-less family members (Zhang *et al.* 2006, 2009). Out of the four newly discovered *Arabidopsis SOFLs*, only *AtSOFL5* and *AtSOFL6* contain introns, a departure from the previous designation of the *Arabidopsis SOFL* family as being comprised of members lacking introns (Zhang *et al.* 2006, 2009). Genes lacking introns are a characteristic of prokaryotes and are a useful resource for studying the evolution of gene architecture in eukaryotes, but information on their biological significance remains limited (Yan *et al.* 2014). Considering that *SOFLs* seem to have emerged during the evolution of angiosperms, it is surprising that the majority of the genes in this family are intron-less, a trait expected in early species (Zou *et al.* 2011). On the other hand, this result can be explained by the fact that majority of the intron-less *SOFLs* could have potentially arisen because of gene duplication events (Yan *et al.* 2016; Lecharny *et al.* 2003). Gene prediction and gene functional studies data suggests that intron-less genes may play unique roles in growth and development, including translation and energy metabolism in maize, rice and *Arabidopsis* as well as cell envelope and amino acid biosynthesis in rice and *Arabidopsis* (Yan *et al.* 2014; Jain *et al.* 2008). In addition, gain-of-function studies from Zhang *et al.* (2006, 2009) suggested that the three-founding intron-lacking *SOFLs* may be involved in CK-related functions. According to Zhao *et al.* (2014) the presence of introns enhances the transcription of associated genes. Therefore, to further explore the biological significance of intron-less genes, studies that include gain-of-function, loss of function, gene expression pattern, gene expression level analysis and subcellular localization will need to be performed in the future.

### SOFL-A and SOFL-B domains are important for SOB5 and AtSOFL1’s overexpression phenotypes

Previously, Zhang *et al.* (2009) demonstrated via site-directed mutagenesis experiments that certain amino residues in the SOFL domains were necessary for the manifestation of *AtSOFL2’s* overexpression phenotype. In our study, we have similarly shown that specific conserved amino acid residues in SOFL-A and SOFL-B domains are also necessary for both *SOB5′s* and *AtSOFL1’s* overexpression phenotypes (Figure 4). These results, together with sequence logo analysis showing that certain amino acid residues in SOFL-A and SOFL-B domains are 100% conserved in all 289 SOFLs, further strengthen the hypothesis that they are important for function. These results should, however, be interpreted with caution because point mutations can potentially cause proteins to fold incorrectly. Nonetheless, our results demonstrated, at least, that not all point mutations lead to a loss of protein function. Future experiments in which all conserved residues are replaced with alanines or entire domains are deleted may provide answers to whether all conserved residues in SOFL-A and SOFL-B domains are important for function. It is not yet clear what biological role these highly conserved domains play. However, conserved domains typically play crucial roles in protein-protein interactions, DNA binding, and other important cellular processes.

### Founding Arabidopsis thaliana SOFL members localize to the nucleus and cytoplasm

To begin to examine the intracellular distribution of SOFLs homologs we first used nuclear localization signal (NLS) detection and sub-cellular localization programs, SeqNLS (Lin and Hu 2013) and Wolf PSORT (Horton *et al.* 2007). The two programs predicted that majority of SOFLs contained NLSs and may localize to the nucleus among other cellular locations and organelles (Table S3). This hypothesis was further supported by CFP-SOFL protein fusion subcellular localization experimental data, which showed that SOB5, AtSOFL1 and AtSOFL2 localize to the nucleus and the cytosol (Figure 5). However, SOB5 subcellular localization results were at odds with findings by Zhang *et al.*, (2006) who reported that SOB5 only localized to the cytoplasm and/or plasma membrane, but not the nucleus. These contrasting conclusions could be due to a different experimental design and fluorescence detection methods used in both studies. The SOB5-GFP C-terminal fusion protein in Zhang *et al.* (2006) was missing five C-terminal amino acids from SOB5, whereas in our case we used a full-length SOB5 protein fused to the carboxyl end of the CFP tag. Second, we used confocal microscopy to detect CFP fluorescence, which is more reliable compared to fluorescence microscopy used by Zhang *et al.* (2006). In addition, we generated and analyzed N-terminal fusions in contrast to the C-terminal fusion described in Zhang *et al.*, (2006). C-terminal and N-terminal tagged proteins have been shown to display opposite localization patterns (Palmer and Freeman 2004), a possibility that may also explain SOB5-GFP and CFP-SOB5 localization pattern. In the future, both C-and N-terminal fusions of each protein should be examined to avoid such potential problems.

Even though we used a plasmolysis assay to show that the three founding SOFLs were not localized to the cell wall, we could not distinguish whether they were localized in the plasma membrane, cytosol or both. To try and answer this question, we used a bioinformatics approach by running the 289 SOFL homolog sequences against web-based transmembrane protein topology prediction algorithms. TMHMM (Kahsay *et al.* 2005) and TMMOD (Kahsay *et al.* 2005). Both hidden Markov prediction models, did not predict any transmembrane helices in all 289 SOFL ortholog sequences. This outcome could be verified experimentally by discriminating between cytosolic and cell membrane proteins using osmotic disruption of the protoplast vacuole in hypotonic solution (Serna 2005). This method results in the diffusion of the GFP signal from the cell periphery to the central part of the cell volume, an outcome that will not occur when the protein under study is attached to the cell membrane (Serna 2005). Overall, these data suggest that at least, the three founding *Arabidopsis thaliana* SOFLs and possibly several other SOFL homologs may localize to the nucleus and/or cytoplasm.

### Conclusion

The identification of at least 289 *SOFL* homologs creates an opportunity for the study and characterization of this novel gene family in at least 89 flowering plant species. A combination of gain-of-function, loss-of-function, protein-protein interaction and CK quantitation studies will go a long way toward answering various questions regarding the function of *Arabidopsis SOFLs* raised by Zhang *et al.* (2006, 2009). One critical question is whether CK nucleosides and nucleotides have biological activity, as suggested by Zhang *et al.*, (2006, 2009). Gain-of-function and CK-quantitation studies in selected species may be used to test the hypothesis that overexpression of *SOB5*, *AtSOFL1* and *AtSOFL2* homologs can cause similar CK-related phenotypes reported by Zhang *et al.*, (2006, 2009). Results from such studies can then be compared to data from higher order null mutants in which putative redundant *SOFLs* from the same phylogenetic clades are knocked out. In addition, our site-directed mutagenesis studies suggested that conserved SOFL-A and SOFL-B domains were important for function. Similar studies involving mutagenesis of additional conserved amino acid residues in both *Arabidopsis* and other species will likely provide new functional details regarding these domains. Finally, even though our latest database searches suggest that *SOFLs* are a plant-specific gene family, the ever-increasing number of sequenced genomes will continue to test this hypothesis.

## Supplementary Material

Supplemental Material is available online at www.g3journal.org/lookup/suppl/doi:10.1534/g3.118.200040/-/DC1.

## References

[bib1] Clough S. J. Bent A. F., 1998 Floral dip: a simplified method for Agrobacterium-mediated transformation of Arabidopsis thaliana. Plant J. 16(6): 735–743. 10.1046/j.1365-313x.1998.00343.x 10069079

[bib2] Crooks G. E. Hon G. Chandonia J. M. Brenner S. E., 2004 WebLogo: a sequence logo generator. Genome Res. 14(6): 1188–1190. 10.1101/gr.849004 15173120 PMC419797

[bib3] Earley K. W. Haag J. R. Pontes O. Opper K. Juehne T., 2006 Gateway-compatible vectors for plant functional genomics and proteomics. Plant J. 45(4): 616–629. 10.1111/j.1365-313X.2005.02617.x 16441352

[bib4] Fang H. Oates M. E. Pethica R. B. Greenwood J. M. Sardar A. J., 2013 A daily-updated tree of (sequenced) life as a reference for genome research. Sci. Rep. 3(1): 2015 10.1038/srep02015 23778980 PMC6504836

[bib5] Goodstein D. M. Shu S. Howson R. Neupane R. Hayes R. D., 2011 Phytozome: a comparative platform for green plant genomics. Nucleic Acids Res. 40(Database issue): D1178–D1186. 10.1093/nar/gkr944 22110026 PMC3245001

[bib6] Guo Y. L., 2013 Gene family evolution in green plants with emphasis on the origination and evolution of Arabidopsis thaliana genes. Plant J. 73(6): 941–951. 10.1111/tpj.12089 23216999

[bib7] Higuchi M. Pischke M. S. Mahonen A. P. Miyawaki K. Hashimoto Y., 2004 In planta functions of the Arabidopsis cytokinin receptor family. Proc. Natl. Acad. Sci. USA 101(23): 8821–8826. 10.1073/pnas.0402887101 15166290 PMC423279

[bib8] Horton P. Park K.J. Obayashi T. Fujita N. Harada H., 2007 WoLF PSORT: protein localization predictor. *Nucleic Acids Res* 35 (Web Server issue):W585–587. 10.1093/nar/gkm259 PMC1933216 17517783

[bib9] Jain M. Khurana P. Tyagi A. K. Khurana J. P., 2008 Genome-wide analysis of intronless genes in rice and Arabidopsis. Funct. Integr. Genomics 8(1): 69–78. 10.1007/s10142-007-0052-9 17578610

[bib10] Kahsay R. Y. Gao G. Liao L., 2005 An improved hidden Markov model for transmembrane protein detection and topology prediction and its applications to complete genomes. Bioinformatics 21(9): 1853–1858. 10.1093/bioinformatics/bti303 15691854

[bib11] Kersey P. J. Allen J. E. Armean I. Boddu S. Bolt B. J., 2016 Ensembl Genomes 2016: more genomes, more complexity. Nucleic Acids Res. 44(D1): D574–D580. 10.1093/nar/gkv1209 26578574 PMC4702859

[bib12] Kim S. Y., 2006 The role of ABF family bZIP class transcription factors in stress response. Physiol. Plant. 126(4): 519–527.

[bib13] Kim S. Y. Kim S. G. Kim Y. S. Seo P. J. Bae M., 2007 Exploring membrane-associated NAC transcription factors in Arabidopsis: implications for membrane biology in genome regulation. Nucleic Acids Res. 35(1): 203–213. 10.1093/nar/gkl1068 17158162 PMC1802569

[bib14] Kondou Y. Higuchi M. Matsui M., 2010 High-throughput characterization of plant gene functions by using gain-of-function technology. Annu. Rev. Plant Biol. 61(1): 373–393. 10.1146/annurev-arplant-042809-112143 20192750

[bib15] Lamesch P. Berardini T. Z. Li D. Swarbreck D. Wilks C., 2012 The Arabidopsis Information Resource (TAIR): improved gene annotation and new tools. Nucleic Acids Res. 40(Database issue): D1202–D1210. 10.1093/nar/gkr1090 22140109 PMC3245047

[bib16] Lecharny A. Boudet N. Gy I. Aubourg S. Kreis M., 2003 Introns in, introns out in plant gene families: a genomic approach of the dynamics of gene structure. J. Struct. Funct. Genomics 3(1–4): 111–116. 10.1023/A:1022614001371 12836690

[bib17] Lin J. R. Hu J., 2013 SeqNLS: nuclear localization signal prediction based on frequent pattern mining and linear motif scoring. PLoS One 8(10): e76864 10.1371/journal.pone.0076864 24204689 PMC3812174

[bib18] Malek J.A., 2001 Abundant protein domains occur in proportion to proteome size. *Genome Biol* 2 (9):RESEARCH0039. 10.1186/gb-2001-2-9-research0039 PMC56900 11574058

[bib19] Miller M. A. Schwartz T. Pickett B. E. He S. Klem E. B., 2015 A RESTful API for Access to Phylogenetic Tools via the CIPRES Science Gateway. Evol. Bioinform. Online 11: 43–48. 10.4137/EBO.S21501 25861210 PMC4362911

[bib20] Palmer E. Freeman T., 2004 Investigation into the use of C- and N-terminal GFP fusion proteins for subcellular localization studies using reverse transfection microarrays. Comp. Funct. Genomics 5(4): 342–353. 10.1002/cfg.405 18629169 PMC2447460

[bib21] Qu L. J. Zhu Y. X., 2006 Transcription factor families in Arabidopsis: major progress and outstanding issues for future research. Curr. Opin. Plant Biol. 9(5): 544–549. 10.1016/j.pbi.2006.07.005 16877030

[bib22] Roshan U., 2014 Multiple sequence alignment using Probcons and Probalign. Methods Mol. Biol. 1079: 147–153. 10.1007/978-1-62703-646-7_9 24170400

[bib23] Sayers E. W. Barrett T. Benson D. A. Bryant S. H. Canese K., 2009 Database resources of the National Center for Biotechnology Information. Nucleic Acids Res. 37(Database issue): D5–D15. 10.1093/nar/gkn741 18940862 PMC2686545

[bib24] Serna L., 2005 A simple method for discriminating between cell membrane and cytosolic proteins. New Phytol. 165(3): 947–952. 10.1111/j.1469-8137.2004.01278.x 15720705

[bib25] Vollrath D. Jaramillo-Babb V. L. Clough M. V. McIntosh I. Scott K. M., 1998 Loss-of-function mutations in the LIM-homeodomain gene, LMX1B, in nail-patella syndrome. Hum. Mol. Genet. 7(7): 1091–1098. 10.1093/hmg/7.7.1091 9618165

[bib26] Wang Y. Wang X. Paterson A. H., 2012 Genome and gene duplications and gene expression divergence: a view from plants. Ann. N. Y. Acad. Sci. 1256(1): 1–14. 10.1111/j.1749-6632.2011.06384.x 22257007

[bib27] Wheeler D. L. Barrett T. Benson D. A. Bryant S. H. Canese K., 2008 Database resources of the National Center for Biotechnology Information. Nucleic Acids Res. 36(Database issue): D13–D21. 10.1093/nar/gkv1290 18045790 PMC2238880

[bib28] Xie Q. Sanz-Burgos A. P. Guo H. Garcia J. A. Gutierrez C., 1999 GRAB proteins, novel members of the NAC domain family, isolated by their interaction with a geminivirus protein. Plant Mol. Biol. 39(4): 647–656. 10.1023/A:1006138221874 10350080

[bib29] Yan H. Dai X. Feng K. Ma Q. Yin T., 2016 IGDD: a database of intronless genes in dicots. BMC Bioinformatics 17(1): 289 10.1186/s12859-016-1148-9 27465544 PMC4964316

[bib30] Yan H. Zhang W. Lin Y. Dong Q. Peng X., 2014 Different evolutionary patterns among intronless genes in maize genome. Biochem. Biophys. Res. Commun. 449(1): 146–150. 10.1016/j.bbrc.2014.05.008 24820954

[bib31] Zhang J. Vankova R. Malbeck J. Dobrev P. I. Xu Y., 2009 AtSOFL1 and AtSOFL2 act redundantly as positive modulators of the endogenous content of specific cytokinins in Arabidopsis. PLoS One 4(12): e8236 10.1371/journal.pone.0008236 20011053 PMC2785485

[bib32] Zhang J. Wrage E. L. Vankova R. Malbeck J. Neff M. M., 2006 Over-expression of SOB5 suggests the involvement of a novel plant protein in cytokinin-mediated development. Plant J. 46(5): 834–848. 10.1111/j.1365-313X.2006.02745.x 16709198

[bib33] Zhang J. Z., 2003 Overexpression analysis of plant transcription factors. Curr. Opin. Plant Biol. 6(5): 430–440. 10.1016/S1369-5266(03)00081-5 12972043

[bib34] Zhao J. Favero D. S. Qiu J. Roalson E. H. Neff M. M., 2014 Insights into the evolution and diversification of the AT-hook Motif Nuclear Localized gene family in land plants. BMC Plant Biol. 14(1): 266 10.1186/s12870-014-0266-7 25311531 PMC4209074

[bib35] Zou M. Guo B. He S., 2011 The roles and evolutionary patterns of intronless genes in deuterostomes. Comp. Funct. Genomics 2011: 680673 10.1155/2011/680673 21860604 PMC3155783

