# Supplementary material for: Synopsis of the SOFL Plant-Specific Gene Family
Source: G3 (Bethesda). 2018 Feb 23;8(4):1281–90. doi: 10.1534/g3.118.200040 (PMC5873917; doi:10.1534/g3.118.200040)
Supplement: Supplementary file 1 [file 1281FileS1.docx]

**SOFL protein sequences**

>AaSOFL1

MESSKIFASDEDCDRSCESGWTMYLASPSNEDDCYYDDDEEDSDGGDSMDSDASSGPMEDTSCLKLPQEIEEQSSLKKKKTKKANEDMVLVETTRVYNSHHNDDDDDDDGGDDHDYDDGNDSYSAVHSYVRQEGLF

>AaSOFL2

MESPRIHGGDAEEKSSCESGWTIYIEDTFHGNHHSDVVYEDYDDVGDGNDDDDGRVKEVDDDGGDSSDNESDDSMTSDASSWPSTQLPRNTKNHAAAKNSNAKQVNHQTKNRACEAFSDEGEESELKGKTRTTATSRVQSKGKVNRTK

>AcSOFL1

MDASDHVFEEAEECTSNESGWTMYIASPMHDDDDDENDDGDLSDGEEHEDDDSDDSMLSDASSGPSHLQEPLGKVDDGHGFSTSFNNSKKKDDSDRKFYSYNKPNRQMEKKRGEGKNIKLEKREASELVVNSSTSSVLSGTKVRKISWMGNKGN

>AhSOFL1

MESANIFGSDEDGRSCESGWTMYLASQSHDHDDDCYYDDDDEEAEDSDGGDSMDSDASSGPMEATISSLKLAQEIEEQNSIKKKKKRTNEEMVLVETTRVHNTIIDDDDDDDGDNHDYDDGNDSYSVVHSYVGSVIQKGLV

>AhSOFL2

MESPRIHGGAEEKSSCESGWTMYIEDTFHGNHHSEVVYEDDDDGDDGFCVKEVDDEDDDGDGDEDDDDDDDDSSNNESDDSMTSDASSWPSTHQPLRSTKNHAAAKNSNAKQVNHQTENRVRDRFSDEGEESELKARTRTTAASRVKSRGKVSKK

>AhSOFL3

MESPRNHGGSEEEEYSSCESGWTMYIEDAFHGNDHSSVVVDDDDDTLVKEADDGYENDDGDNSDDGGDEESDDSMASDASSGPSNQLPKNINKHAARKNGSKQVYLQKRQHTEKTLSNEGEKSDLKARTRTSAASRVQSRGKVSKTK

>AlSOFL1

MESANIFGSDEDGRSCESGWTMYLASQSHDHDDDCYYDDDDEEAEDSDGGDSMDSDASSGPMEATSSLKLAQEIEEQNSIKKKKKRTNEEMVLVETTRVHNTIIDDEDDDDGDNHDYDDGNDSYSVVHSYVGSVIQKGLV

>AlSOFL2

MDKEECSSSESGWTTYISSPIKVDEDEVVDEDYYEGYNIYNYSSKVEHEEERNKDSDDSMASDASSGPNYQRYHQKNKALDLKKGKNEGNTKSKNVDDHHNHYHDGKNTSNSYRKKEKKKRENKSTYRMK

>AlSOFL3

MESPRNHGGSEEEEYSSCESGWTMYIEDAFHGNDHSSVVVDDDDDDTQVKEAHDGYENDDGDNSDDGGDEESDDSMASDASSGPSNQLPKNINKHAARKNGSKQVYLQKRQHTEKTLSNEGEKSDLKAKTRTSAASRVQSRGKVSKTK

>AlSOFL4

MEREECNSSESGWTTYISSPLEEEEEEVIDEVYYEGHSIEKDRRKFANEYENNKDSDDSMASDASSGPGYQQYPQTNDRGKRREGLAVRNGKGESNNTSNDAYLHHIDDKNSGNHISRKKEKKKIENKSRSHKKK

>AlSOFL5

MDFSDLNDSDAGDSGWTMYLDHSSSVSLHHFDYHNGDTKQEHDDDSSMVSDASSGPPYYCEETVPEDLLQQNTQYWCKSKSKIKNKNKNKNKNKKKVHEEQGYSERFNSSLDDTASSLGKFNSGFLQQAFPVDKLGLDNQGGSNQRKRRG

>AlSOFL6

MLGSSSGCESGWTLYLDQSVSSPTPSCYRDSNGFENRRRSKDSWDQHNVHQGDEEEEEEDDLSMISDASSGPRNISEEDSVNKKINIVGLKKQCKREKKRRDYEKMNSVLDDTASSPLFNFPHMLQKSVGGNKIEQNFPESTLDYSQGFSATQFQDNTAFQEQYGYLHMETRF

>AlSOFL7

MESPRIHGGAEEKSSCESGWTMYIEDTFHGNHHSEVVYEDDDDGDDGFCVKEVDDEDDDGDGDEDDDDNSNNESDDSMTSDASSWPSTHQPPRSTKNHAAAKNSNAKQVNHQTENRVRDRFSDEGEESELKARTRTTAASRVKVSKTK

>AtaSOFL1

MESSQITGDGGEECNSNESGWTIYLTSPTSSYEAKENGSEGSNVEDGSGYITERRKGKEENNADDDGDYDSLASDASTGTSQVKVLEGKEEKDRQTNHGSSNEHVKDEQAETLTKFSTGSNKKAGKVKKGDEKSSKRGHNRRRSSSRTSFFW

>SOB5

MESANIFGSDEDCRSCESGWTMYLASQSHDRDDDCYYGDDDEEEEDSDGGDSMDSDASSGPMEATSYLKLAQEIEEQNSIKKKKKKTNEEMVLVETTRVHNNINHDDDDDDDEDDGDNHDYDDGNDSYSVVHSYVGSVRQKGLV

>AtSOFL1

MESPRNHGGSEEEEYSSCESGWTMYIEDAFHGNDQSSVVVDDDDDDTQVKEADDGYENDDGDTSDDGGDEESDDSMASDASSGPSNQLPKHINKHAARKNGSKQVYLQKRQHTEKTISNEGEKSDLKARTRTSAASRVQSRGKVSKTK

>AtSOFL2

MESPRIHGGAEEKSSCESGWTMYIEDTFHGNHHSEVVYEEEDDGFSVKEVDDDGDGDEDDDDDDDDDSSNNESDDSMTSDASSWPSTHQPPRSTKNHAAAKNSNAKQVNNQTENRVRDRFSDEGEESELKARTRTTAASRVKVSKTK

>AtSOFL3

MEREECSSSESGWTTYISSRMEEEEEEVIDEVYYEGHIIEKDRRKYANEYEINKDSDDSMASDASSGPSYHQTSNRGKRREGLALRNGKGESNDVYSHRIDDKNIGNLISRKKEKKKSENKSRSHKKK

>AtSOFL4

MDKEECSSSESGWTTYLSSPIKVDEDEVVDEDYYYEGYNIYNYSSKVEHEEERNKDSDDSMASDASSGPNYQRFHQKNKALDLKNGKNEGNSKSKNDDDHHNHYHDGKKTSNSYRKKDKKKRENKSTYRMK

>AtSOFL5

MLGSSSGCESGWTLYLDQSVSSPSPSCFRDSNGFDSRRRSKDSWDQNYVHQEEEEEEDDLSMISDASSGPRNISEEDSVKKINIVGLKKQCKREKKRRDYEKMNSLLDDTASSPLFNFPHMLQKSVGGNKIEQTFPESTLDYSQGFSATQFQDKTAFQEQCGYLHMETRF

>AtSOFL6

MDFSDLDYSDAGDSGWTMYLGHSSSVSLHHFDYHNGETKQEHDEDSSMVSDASSGPPYYCEETVHEDHLQQNTQYWCKSKSKNKNKNKKKVHEEQGYSERFNSSFDDTASSLPVGEEVSAHKQHQNQYQRFHDFCQSYSTRRICKEKVNSGFLQQAFPVDKLALDNQGGDNQRKRRG

>BdSOFL1

MESSHITGDDGEGCNSSESGWTMYLSSPMQGDDDDGNGGGKGSGSDGSNVDDGYGYTYIVHGRKGGKEYQDDGDDNDSLASDASTGPAKVKSPPCLPNANGKEDHPSPRRATDEDRKEEDDDEEEDDGRRSRFSTSSRKKAGKVEKGGGGDAKSSSKRGHGKRGSSSRTSFFW

>BdSOFL2

MESSQITGDDGEECSSNESGWTIYLASPMRSDDVKENGSEGSNVEDGSGYINERRKGKEDHNQDDDGDYDSLASDASTGPAQVKALEGKEEKNRQTNVGCSDEQGKDEQDEIRTKILTTCNKKAGKIKKGDEKTSRRGHSKRRSSSRTSFFW

>BnSOFL1

MDSGKIFGSDEDSRSCGESGWTTYLVSTHDHDYDNYSDDGDSSGGDSMDSDASSGPVKATPCLKLAQETTEPNCLKKKKATEEKVLVETRVHNDNDDDGDNHDYDDGDNHDYDDGDNHDYDDGNDSHSAVQSYVGSV

>BnSOFL2

MESGKSFGTEDVSRSCESGWTMYLASHSHDPDDDCYYEDGDDEDSDGGESMDSDASSGPMEATSTLKLPQDTEEQNSIKKKKKANEEMVLVETRVDNNNHDDDAYDNDDVYNHDDGNDSYSAVHSYVGAVRQGQAAREKTQWEAKTLGERETKLEDNLDSLQGDISSFTRDDVDETELRRLEDARLAYVAAVSNAKERQDEESLAMAAKARAYLQSLAFRY

>BnSOFL3

MESGKSFGTEDVSRSCESGWTMYLASHSHDPDDDCYYEDGDDEDSDGGESMDSDASSGPMEATSTLKLPQDTEEQNSIKKKKKANEEMVLVETRVDNNNHDDDAYDNDDVYNHDDGNDSYSAVHSYVGAVRRGFV

>BnSOFL4

MESGKIFGSDEDSRSCESGWTMYLASLSDDHDQECYYDDDGDEDSDGGDSMDSDASSGPMEATANLKLTQEIAEQNSTKKKNKKTIEDTVLVDTRVHNNNHDDDDDGRDNHELDHDDGNDSYSAVHSYIGSVRQEGLI

>BnSOFL5

MESGKIFGSDEDSRSCESGWTTYLASLSNDHDQDCYHDDDGDEDSDGGDSMDSDASSGPMEATANLKLPQEIAEQNSIKKKNKKTNEEMILMETRVHNNNHDDGDDHEFDDDDDDGNDSYSAVHSYVGFRLDKKN

>BnSOFL6

MESPRDHGGSEEEEEYNSCESGWTMYIEDAFGGNGHSSIVVVDDDDDPQVKEADDGGDEESDDSMASDASSGPSNQLLKNINKHAARENVSKKVYIQKRQHTEKTLSQEEEKSEVKARTRTSAASRVQSKGKVSKTK

>BnSOFL7

MESPRDHGGSEEEEEYNSCESGWTMYIEDAFGGNDHSSIIVDDDDDDDSQVKEADDGGDEESDDSMASDASSGPSNQLPKNINKHAARKNVSKQVYIQKRQHTEKTLSKEEEKSELKARTRTSGASRVQSKGKVSKTK

>BnSOFL8

MESPRNHGASEEEEYSSCESGWTMYIGDAFQGNGHSTVVVDDDGDDDDEYSHVKDVDDGYENNDGGKESDDSMASDASSGPSNQISNNINKHAARKNGSKQVCIQKRQPTEKTLSNEGEKSEVKARTRTSAASRAQSRGKVSKTK

>BnSOFL9

MEREECSSSESGWTTYISSPMEDDEQEVTDEVYYEGHITGNDQRKHVNEYENNKDSDDSMASDASSGPSYPQTSNRGRSRECIVLRNGKSESKSKSKSNEDYDHKHNAKNSDNYKCRIKEKKKIESKSKSFKKK

>BnSOFL10

MEREECSSSESGWTTYISSPMEEDEEEVIDEVYYEGHSIEKDGRKYVNEYESNKDSDDSMASDASSGPSYHQTSNRGRRREGLVLRNGKSESNSKSNSVYDYKHDDKNSGNHKSRTKEKKKGENKSRSSKKK

>BnSOFL11

MESPSIHGGSEEKSSCESGWTMYIEDTFHGSHHSEEYNDDDGDDFRVKEVDDDSSKNGSDDSMTSDASSWPSTQLPRNTKNHAEAKNSNAKKVNHQTKNRASEKSSNQEEESEFKARTRTTAASRVRSRDKVSKTK

>BnSOFL12

MEREECSSSESGWTTYISSPMEEDEEEVIDEVYYDGHSIEKDGRKYVNEYESNKDSDDSMASDASSGPSYHQTSNRGRRREGLVLRNGKSESNSKSNNVYDYKHDDKNSGNHKTRTKEKKKGENKSRSSKKK

>BnSOFL13

MESPRRGGSEEKSSCESGWTMYIEDTFHGNHHSEVVYEEYDDDDDFHGKEVDDDGDGDDDSSENGSDDSMTSDASSWPSTQQPRNPKNHAAAKKSNAKQVSHQTKNRACEKFSDEEEESEFKARTRTTTTSRVQSRHKASKTK

>BnSOFL14

MESPRRGGSEEKSSCESGWTMYIEDTFHGNHHSEVVYEEYDDDDDFNGKEVDDDGDGDDDSSENGSDDSMTSDASSWPSTQHPRNPKNHAAAKKSNAKQVSHQTKNRACEKFSDEEEESEFKARTRTTTTSRVQSRGKASKTK

>BnSOFL15

MESPRIHEGVAEEKSSCESGWTMYIEDTIHGNHHSEFVYEEYDDDGNHFCVKDVDDDSSENGSDDSMTSDASSWPSTQLPRKTKNHAAAKKSYAKQVNHHTKNKAREKSTDQEEESEFNGRTRTIAASRVQSKGKDYVFGLKQIPFL

>BnSOFL16

MESPRIHEGDAEEKSSCESGWTMYIEDTFHGNHHSEFVYEEYDDDGNHFRVKDVDDDSSENGSDDSMTSDASSWPSTQLPRKTKNHAAAKKSNNAKQVSHHTKSRAREKSTDQEEESEFKGKTRTIAASRVQSKGRVRKNK

>BnSOFL17

MESPRNHVGSEEEEYNSCESGWTMYIGDAIQGNDHSTVVVDDNDDDESHVKYADDGYENDDGGKESDDSMTSDASSGPSNHLPNNINKHAARKNGSKQVYIEKRQPKEKTLSNEGEKSEVKARTRTIAASRVQSRGKVSKTK

>BnSOFL18

MESPRIHEGVAEEKSSCESGWTMYIEDTFHGNHHSEFVYEEYDDDGNHFRVKDVDDDSSENGSDDSMTSDASSWPSTQLPRKTKNHAAAKKSNNAKQVSHHTKSRAREKSTDQEEESEFKGRTRTIAASRVQSKGRVRKNK

>BnSOFL19

MESPRIHEGVAEEKSSCESGWTMYIEDTIHGNHHSEFVYEEYDDDGNHFCVKDVDDDSSENGSDDSMTSDASSWPSTQLPRKTKNHAAAKKSNAKQVNHHTKNKAREKSTDQEEESEFNGRTRTIAASRVQSKGKVRKNK

>BnSOFL20

MESPRIHEGVAEEKSSCESGWTMYIEDTIHGNHHSEFVYEEYDDDGNHFCVKDVDDDSSENGSDDSMTSDASSWPSTQLPRKTKNHAAAKKSYAKQVNHHTKNKAREKSTDQEEESEFNGRTRTIAASRVQSKGKVRKNK

>BnSOFL21

MESGKIFGSDEDSRSCESGWTMYLASLSDDHDQDCYYDDDGDEDSDGGDSMDSDASSGPMEATANLKLTQEIAEQNSTKKKNKKTIEDTVLVDTRVHNNNHDDDDDGRDNHELDHDDGNDSYSAVHSYIGSVRQEGLI

>BnSOFL22

MDSGKIFGSDEDSRSCGESGWTKYLVSTHDHDYDNYSDDGDSSGGDSMDSDASSGPVKATPCLKLPQETTEPNCLKKKKATEEKVLVETRVHNDNDDDGDNHDYDDGDNPDYDDGDNHDYDDGNDSHSAVHSYVGSV

>BrSOFL1

MESGKSFGTEDVSRSCESGWTMYLASHSHDPDDDCYYEDGDDEDSDGGESMDSDASSGPMEATSTLKLPQDTEEQNSIKKKKKANEEMVLVETRVHNNNHDDDAYDNDDVYNHDDGNDSYSAVHSYVGAVRQGFV

>BrSOFL2

MDSGKIFGSDEDSRSCGESGWTTYLVSTHDHDYDNYSDDGDSSGGDSMDSDASSGPVKATPCLKLAQETTEPNCLKKKKATEEKVLVETRVHNDNDDDGDNHDYDDGDNHDYDDGNDSHSAVQSYVGSV

>BrSOFL3

MESGKIFGSDEDSRSCESGWTTYLASLSNDHDQDCYHDDDGDEDSDGGDSMDSDASSGPMEATANLKLPQEIAEQNYIKKKNKKTNEEMILMETRVHNNNHDDGDDHEFDDDDDDGNDSYSAVHSYVGFRLDKKN

>BrSOFL4

MESGKSFGTEDVSRSCESGWTMYLASHSHDPDDDCYYEDGDDEDSDGGESMDSDASSGPMEATSTLKLPQDTEEQNSIKKKKKANEEMVLVETRVHNNNHDDDAYDNDDVYNHDDGNDSYSAVHSYVAAVRQGFV

>BrSOFL5

MESPRNHVGSEEEEYSSCESGWTMYIGDAIQGNDHSTVVVDDNDDDESHVKYADDGYENDDGGKESDDSMTSDASSGPSNHLPNNINKHAARKNGSKQVYIEKRQPKEKTLSNEGEKSEVKARTRTSAASRVQSRGKVSKTK

>BrSOFL6

MESPRNHVGSEEEEYSSCESGWTMYIGDAIQGNDHSTVVVDDNDDDESHVKYADDGYENDDGGKESDDSMTSDASSGPSNHLPNNINKQAARKNGSKQVYIEKRQPKEKTLSNEGEKSEVKARTRTSAASRVQSRGKVSKTK

>BrSOFL7

MESPRNHGASEEEEYSSCESGWTMYIGDAFQGNGHSTVVVDDDGDDDDEYSHVKDVDDGYENNDGGKESDDSMASDASSGPSNQISNNINKHAARKNGSKQVCIQKRQPTEKTLSNEGEKSEVKARTRTSAASRAQSRGKVSKTK

>BrSOFL8

MESPSIHGGAEEKSSCESGWTMYIEDTFHGSHHSEEYNDDNGDNFRVKEVDDDGDDDSSKNGSDDSMTSDASSWPSTQLPRNTKNHAEAKNSNAKKVNHQTKNRASEKSSNQEEESEFKARTRTNAASRVRSRDKVSKTK

>BrSOFL9

MESPRIHEGDAEEKSSCESGWTMYIEDTFHGNHHSEFVYEEYDDDGNHFRVKDVDDDSSENGSDDSMTSDASSWPSTQLPRKTKNHAAAKKSNNAKQVSHHTKSRAREKSTDQEEESEFKGKTRTIAASRVQSKGRVRKNK

>BrSOFL10

MESPRRGGSEEKSSCESGWTMYIEDTFHGNHHSEVVYEEYDDDDDFHGKEVDDDGDGDDDSSENGSDDSMTSDASSWPSTQQPRNPKNHAAAKKSNAKQVSHQTKNRACEKFSDEEEESEFKARTRTTTTSRVQSRGKASKTK

>BrSOFL11
MESPRIHEGVAEEKSSCESGWTMYIEDTIHGNHHSEFVYEEYDDDGNHFCVKDVDDDSSENGSDDSMTSDASSWPSTQLPRKTKNHAAAKKSYAKQVNHHTKNKAREKSTDQEEESEFNGRTRTIAASRVQSKGKVRKNK

>BsSOFL1

MESANIFGSDEDCRSCESGWTMYLASQSHNHDDGCYYDDDEEEEDSDGGDSMDSDASSGPMEATSCLKLAQEIEEQNSVMKKKKKTNEEMVLVETTRVHNNINDDDDDDIDGDNHDYDDGNDSYSVVHSYVGSVRQKGLV

>BsSOFL2

MESPRNHGGSEEEEEYSSCESGWTMYIEDAFHGNDHSSVVVDDDDDTQVKEGDDGYENVDGDSSDNGGNEESDDSMASDASSGPSKQFPKNINKHATRKNGSKQVYLQKRQHTEKTFSNEGEKSDLKARTRTSAASRVQSRGKVSKTK

>BsSOFL3

MESPRIHGGAEEKSSCESGWTMYIEDTFHGNHHSEVVYDDDDDDDFHVKEVDDDGDDDDDDNSNNESDDSMTSDASSWPSTQPSRSTKNHVAATKSNAKQVNHQTENRVRDRFSDEGEESELKARTRTTAASRVKSRGKVRKTK

>BstSOFL1

MESSHITGDDGEGCNSSESGWTMYLSSPMQGDDDDDDGNGGGKGSGSDGSNVDDGGYGYTYVVHGRKGGNKEYQDDGDDNDSLASDASTGPAKVKPPPCLPNGKQDQGHPSPRRATDEDRKEEDDDEEEDDGRRSRFSTSSRKKAGKAEKGGGGGDAKSSSKRGHGKRGSSSRTSFFW

>CanSOFL1

MEYPKHPGGTEECNSNESGWTMYIGSSYCDYNQGDDDDDNEGTPRKGYKNADDGGSDDSMTSDASSGPSHQGVCTNIEQSYGKYEIKHAEKDTRKFSSKEQQKLAKKKLSDKNTKAAKEDSGHKAKSGKGYGYCRSTTRGKHAN

>CanSOFL2

MEDMSKEECNSNESGWTMYIGSSYCDYNQGDDDDDNEGTPRKGYKNADDGGSDDSMTSDASSGPSHQGVCTNIEQSYGKYEIKHAEKDTRKFSSKEQQKLAKKKLSDKNTKAAKEDSGHKAKSGKGYGYCRSTTRGKHAN

>CarSOFL1

MKPYSKNFGIEEECQSSESGWTMYIGSPIEDDDNDDDDEDDGDIDNMDDEEGTHEAHDDLESDDDSMASDASSGPSHQHYGRDYGLEGLKQVVVEDENNKYCLEKKENKTMENEGKNVEKKEMIFVDGKGKSSVHVGGGKVRKNYLVGGKKGV

>CarSOFL2

MEPPHHMLLGGAEECHSSESGWTMYIGSPIHHEDGNYEDEGNNNNKVEFYQRMTQTQVDVEVESDDSMASDASSRPSHYINVVNSLGSCEGGYGLRHFKQNVEENNQFDHDVAHEYYCLDHVKKGSNKKENQIGETKGEKKLILKGSAQGGGGGGRVKVRKVQRVGTRK

>CarSOFL3

MDSFKQIWGVEGCSSSESGWTMYIDSPLQEDDARCSNENDGYREIYGKNWRRKQGGSKVDEEESDDSMASDASSGPIHYNQQQHGYERGQGSHETSVSNSNSKKDRKDHGSKCSSKKNGNKQEKKRVDSRSKK

>CarSOFL4

MEPSQDLVASGGEFNGTESGWTNYIGSPFHREIYTDEDQSVVDMDEYGNNDEKIHYDNDNKGEKNDENEESDDDSMASDASSGPSYLQLVCIKNERSNSLHEHEKILSTKRVTKQVKKTKKYEGLVAKQEESLLVADSAASHV

>CarSOFL5

MDMSNSQYNSESESGWTNYFNHSSFSEKHFHRKSGKVEYEGKGATMEEEEEEEDLSMVSDASSGPPHYHVEEDYQQQPYCVNWHSSSSKESKKKVKEYGRKSQQPSPLDDTASSPVLNYPKMKVNFPGNGAVENTLDFPPSFSATRIIKQRKTKLQKHSSFLERSLGGKQASEEPGGCNEEERK

>CarSOFL6

MDISTSQYSDASESGWTKYLDQSTLSKSYFHGRDVKTEQDLSMVSDASSGPPHYDNNEYYCENLCPSLSSTIKESKKKKKKVKEYGKSQQPSPLDDTASSSFFSCPKKSHKQESFTGNGAVESALNFSESKFATRIKRKTKF

>CarSOFL7

MSAFDSECSSGCESGWTLYLEHSFGGGYYGEHKDKIVTNDYSEGVEDLSMISDASSGPPQYLPFDDDANYFNQKNNACFYSESNLVKQNKSASKKQNVKDKNKQQLQVEDQQHLPSFLHDTASSHVFDFSTNNDVGTNQQTYKESMVDYSQGFSATNYFEGRSSYQEQHFGYLQPSLSKNEIQGNKWYGGKGMMRGRRC

>CcSOFL1

MDSFKQIWGAEGCSSSESGWTMYIASPMQEDDAGCSNENDGYHDIYGENRRKKQGVKVDEEESDDSMASDASSGPVHYHHAYAHRQSSHGSAASKKDKQDHGSKCSSKKNANKQEKKRVDSRSKK

>CcSOFL2

MEPPHVMLGSSEEECHSNESGWTMYIGSPRDEDDAHCGDEENDRSMDYDYEGAQADPDAESDDSMASDASSGPSHYGTINPLGNGFTNFEPKEEVESDHKASKSKVEEERVVEKNETLFINSQGKAPHQVQNGSKVKVNKNHRVGNRK

>CcSOFL3

MNASASECSSGCESGWTLYLEHSFQLNYNSQFIGEGDDGFCDDKKGKKEEAEEEDLSMVSDASSGPPHLPDAQDNGSLYSASKVAMANLGNKRSKKRQKVKENQHLPSFLDDTASSPFFDFSMNNVTLTNQQTSTESMLDYSQGFSATYYEERSSLQDHLGFLQPSLSENGVHNNK

>CcaSOFL1

MDDPSENIGGREECNSSESGWTMYIASPVHENNPENDDDDDDNYTERKGYKDYPSDDGGDPASDDSMASDASSGPSHQGGPCRTKEGSHRRDDIAHAEGKVNNRRSSGKKHDKQVERKQYAADTKAAKKEQGHKGKNATENLQGKGKSRKN

>CcapSOFL1

MESSKIFGGNEECHSSESGWTMYIGSPIQGGDDDGDHGDSEEAAAVLNANNGGRANETADHDSDDSMASDASSGPSHYGNMVLGLRHGHGTSCFKHEDEDEEGNSKFLDKKATKKSMEKQNFGMKKKGETEEMTFKAKGATTTNITPRSSSKVLEVAISINKSSRAPPEARSISIDGCKIWLFSIKMTSIFLSPYGKERKMYLPMNLGFADQKLAITSCPCSPAFGDPA

>CcapSOFL2

MEASQILSGIEDEEHSSSESGWTMYIGSSIHQTDQYNYIGEFDCDTHKPEEGYRKNRCYNYEGNRKNDDESDDSMASDASSGPSNYKLPCRSEQNLVMDHYKHETFESTTTEKLHKQVMKKDKRRNKMEKEKLELKTISAGSHVGRRDKTK

>CcarSOFL1

MEFSKYLGVAEGCSSSESGWTMYIVSPMHETNNNHHHHDVDDAIDEIHDGYHNGDSDDSMVSDASSGPSRQGILRGSSKRRSGLRRDQSKHAMNKDLIGKNQRQVEKLYERRQRAGKEEEYKNPNDNKARKTS

>CClSOFL1

MNALASECSSGCESGWTLYLEHSSHRNASGRSGGFCHENYYEEKKYSVKHNEHVVDDDEEDLSMVSDASSGPPHFNEDEEVGYFNDVKATTKLPNNGGKRLKNKEQRCREKEKLPCFLDDTASSPVINFSSKNDFALTNTNQASMESMLDYSQAFSATHFQGRSAIFQDRFGFLQPSLSGNHLQNNQWC

>CClSOFL2

MNLSASQCKCSSSGSGCESGWTSYLDQASLSRNRYQCYSLGDGHNEEEEEDDLSMVSDASSGPPHYCEHDEDCFDEKGSFYSPHLPSELAPAKSKNKKKIKQQQHNSYLDDTASSPVKKNISFSKNEASVEQVLGYSQGFSTTHLRGKSTFKKHFDFLPSSLAGKKC

>CfSOFL1

MESSQILGVTEEYGSSESGWTMYIGSPVHGNDSCDADDDHSTDKQGHDHDNDDDYPDNDHNKYESDDSMASDASSGPRHQEFLCESSERSLNINYFKHEASKRTSEKKLCRQVKKRDDRRMKEKQESLHKENVDASHVQGGSNVRKTN

>CgSOFL1

MESANMFGSDEDSRSCESGWTMYLASQSCDHDDGCYYDIDDDEEEEDSDGGDSMDSDASSGPMEATSCLKLAQEIEEQNSAVMKKKKKKTSEEMVLVEATRVHNNINHDDDDDNIGGDDHDYDDGNDSYSVVHNYVGSVRHKGLV

>CgSOFL2

MESPRNHRGSGESEEYSSCESGWTMYIEDAFHGNDHSSVVVDDDDDDTQVKAAYDGYENDDGDSSDDGDNEESDDSMASDASSGNSKQFPKNTSKHAARKNGSKQVYLQKRQHTDKTFSNEGEKSDLKARTRTSAASRVQSRGKVSKTK

>CgSOFL3

MESPRIHGGAEEKSSCESGWTMYIEDTFHGNHHSEVVYDDDDDDDDFHVKEVEDDEDDDDGDDSRNNESDDSMTSDASSWPSTHQPPRNTKNHVAAKNSNTKQVNHQTENNRVRSRFSDQGEESELNARTRTTQASRVKSRSKVSKTK

>CgSOFL4

MAREECSSSESGWTTYISSPMAEDEEEVIHEVYYEKQNIEKDRRRYANEYESNKDSDDSMASDASSGPSYQQYHQTSNRGKRREGLGLRNGKGESNSKSNGVYDHYIDDKNSGNHISRKNEKKKSENKVRYHKK

>CgSOFL5

MEKEECSSSESGWTTYISSPIEVDEEEVVDQDYQEVYNMYNYFSKGETEEERNKDSDDSMASDASSGPNYYQRYHQKNKALELKNGKIEGNNTKSKNDIDKKTSNSYKKKEKKKRETKSTYRK

>CmSOFL1

MEPSQIFGVSEECHSSESGWTMYIGSPANDDSSDAASDDDDKDEEHKGYYYAANNHNDSDDDSMASDASSGPSHQKGNHNPFKGMKNPNGEMNFCLETTRTVRKPLMEKKKKQRAERKEVKVGQKPKTSVQSSSKVRKNILMSKRN

>CoSOFL1

MESSKIFGGNEECHSSESGWTMYIGSPIQGGGDDDGDDGESEEAAAVFNGNNGGRANGTADHDSDDSMASDASSGPSHYGNMEVGLRHGTSCFKHENEDDEEEEGNSKFLDKKATKKSMEKQNFGMKKKGEIKEEMAFKAKGATTTNITPRSSSKVRKSIWLGKRK

>CoSOFL2

MEASQILSGVEDEEHSSSESGWTMYIGSSIHQTDQYNYIGEFDCDTHKPEEGYRKNHCYNYEGNRKNEDESDDSMASDASSGPSNYKLPCCSEQNLVMDHYKHEPFKSTTTEKLHKQVMKKEKRRNKMEKEKFELKTISAGSHVRSRDKTK

>CpSOFL1

MESSKILGGAEEDSSSESGWTMYIDSSSHEVYDSDQDNQSTDKHTYDYRKDNRYNNNQNDDDNESDDSMASDASSGPSHQLSWGSSTQSLSIGYFKQAATKYPSKQEPNRPVKKKDHRTQAEKSVIKAKSIASHVQSGTKMRKTN

>CpSOFL2

MESSKNLSGGTEGCSSSESGWTTYIASPMEEDDAECGEDEDGNYENDNDDANDAENDNGGQDSDDSMASDASSGPSHRQQKHEKDKGSHGRVHSKHGKGDYVSKCSSHNKNSKQEKKRGENSTRYRVQN

>CrSOFL1

MESPRIHGGAEEKSSCESGWTMYIEDTFHGNHHSEVVYDDDDDDDDFHVKEVEDDDDDDDDGDDSRNNESDDSMTSDASSWPSTHQPPRNTKNHVAAKNSNTKQVNHQTENNRVRSRFSDQGEESELNARTRTTQASRVKSRSKVSKTK

>CrSOFL2

MAREECSSSESGWTTYISSPMAEDEEEVIHEVYYEKQNIEKDRRRYANEYESNKDSDDSMASDASSGPSYQQYHQTSNRGKRREGLGLRNGKGESNSKSNGVYDHYIDDKNSGNHISRKNEKKKSENKVRYHKK

>CsaSOFL1

MESANIFGSDEDCRSCESGWTMYLASQSHDRNHDGCYYDDDDEEEEDSDGGDSMDSDASSGPMEATSCVKLAQEIEEQNSVMKKKKKTNEEMVLVETTRV

>CsaSOFL2

MESANIFGSDEDCRSCESGWTMYLASQSHDRNHDGCYYDDDDEEEEDSDGGDSMDSDASSGPMEATSCVKLAQEIEEQNSVMKKKKKTNEEMVLVETTRVHNNVNDDEDDIDGDNHDYDDGNDSYSVVHSYVGSVRQKGLV

>CsaSOFL3

MESANILRSDEDCRSCESGWTMYLASQSHDHNHDGCYYDDDDEEEEEDSDGGDSMDSDASSGPMEATCLKLAQEIEEQNSVMKKKKKTNEEMVLVETTRVHNNVNDDEDDIDGDNHDYDDANDSYSVVHSYVGSVRQKGLV

>CsaSOFL4

MESANIFGSDEDCRSCESGWTMYLASQSHDHNHKGCYYDDDDDEEEDSDGGDSMDSDASSGPMEATSCLKLAQEIEEQNSVTKKKKKTNEEMVLVETTRVHNNVNDDEDDEDDIDGDNHDYDDGNDSYSVVHSYVGSVRQKGFV

>CsaSOFL5

MEREECSSSESGWTTYISSPMEEEVIDELYYEGHSIVKDRSKYANEYESSKDSDDSMASDASSGPSYQXYHQTSNRGKRREGLGLCNEKCESNSKCNDVYDHHTDDKNSGNRISRKTEIKKSENKGRYHKKK

>CsaSOFL6

MESPRNHGGSEEEEEYSSCESGWTMYIEDAFHGNDHSSVVVDDDDDTQVKEADHDGYENDDGDSSDDGGNEESDDSMASDASSGPSKQFPMNINKHAARKNGSKQVYLQKHQHTEKTFSNEGEKSDLKARPRTSAASRVQSRGKVSKTK

>CsaSOFL7

MESPRNHGGSEEEEEYSSCESGWTMYIEDAFHGNDHSSVVVDDDDDTQVKEADHDGYENDDGDSSDDGGNEESDDSMASDASSGPSKQFPMNINKHAARKNGSKEVYLQKHQHTEKTFSNEGEKSDLKARTRTSAASRVQSRGKVSKTK

>CsaSOFL8

MESPRRIHGGAEEKSSCESGWTMYIEDTFHGNHHSEVVYDDDDDDEDDFHVKEVDHDGGDDDDDDDGDDSSNNESDDSMTSDASSWPSTQHPRNNKNHAASTKNSNAKQQQVNHEKENRVRDRFSDQGEESELKARTRTTATSRVKSRSKVSKTK

>CsaSOFL9

MESPRRIHGGAEEKSSCESGWTMYIEDTFHGNHHSEVVYDDDDDDEDDFHVKEVDHDGGDDEDDDDDDDSSNNESDDSMTSDASSWPSSQHPRNTKNHAASTKNSNAKQQQVNHQKENRVRDRFSDQGEESELKARTRTTATSRVKSKSKVSKTK

>CsaSOFL10

MESPRRIHGGAEEKSSCESGWTMYIEDTFHGNHHSEVVYDDDDDDEDDFHVKEVDHDGGDDDDDDDGDDSSNNESDDSMTSDASSWPSSQHPRNTKNHAASTKNSNAKQQQVNHEKENRVRDRFSDQGEESELKARTRTTATSRVKSKSKVNKTK

>CsiSOFL1

MEPSKILGCKEECSSSASGWTMYIGSPAHENDNCEDDDDDDSGTYKQKVASNNDDRPGDDDDDDDKSDDSMASDASSGPSYQELPLDSNKPSLDKHATGKYSSKQKLHKQKTKRDESRIKVERDEQHVLKAKIAASQTQSGAKVRKSK

>CsiSOFL2

MDNSKYPFGTEGCSSSESGWTMYIDSPVQEDMECYNEEDYSNDRKSNYNDGIHGGFNKEDSDDSMASDASSGPSHQHKRANDEGRHSTASSKHGKSGTKKLFSWKKSNEKEKKSVDHSSKHKHKNDAHKKHFK

>CsiSOFL3

MDNSKYPFGTEGCSSSESGWTMYIDSPAQEDDMECYNEEDYSNDRKSNYNDGIHGGFNKEDSDDSMASDASSGPSHQHKRPNDEGRHSTASSKHGKSGTKKLLSWKKSNEKEKKSVDHSSKHKHKNDAHKKHFK

>CsiSOFL4

MEPSKILGCKEECSSSASGWTMYIGSPAHENDNCEDDDDDSGTYKQKVASNNDDRPGDDDDDDDKSDDSMASDASSGPSYQELPLDSNKPSLDKHATGKYSSKQKFYKQKTKRDESRIKVERDEQHVLKAKIAASQTQSGAKVRKSK

>CsiSOFL5

MDSSKVFGGAEECQSNESGWTMYIGDAAAAATTDDGDDDGSDHDGTMNANHEDDYSDDSMASDASSGPSHHHYGTAEALSRPIKEADEDDNSKCTAGSDKKAKRIMEKQKAEMRRKEQEKDHQEMMLMSRRVITPAQSDSKARKNVWNMTKRK

>CsiSOFL6

MNALASECSSGCESGWTLYLEHSSHRNASGRSGGFCEENYYEEKKYSVKHNEHVVDDDEEDLSMVSDASSGPPHFNEDEEVGYFNDVKATTKLPNNGGRRLKNKEQRCREKEKLPCFLDDTASSPVINFSSKNDFALTNTNQASMKSMLDYSQAFSATHFQGRSAIFQDRFGFLQPSLSGNHLQNNQWC

>CusaSOFL1

MEPSQIFGVSEECHSSESGWTMYIGSPANDDSSDAASDDDDEDEEHKGYYYAANNHNDSDDDSMASDASSGPCHQKGNHNPFKGMKNPNGEMNFCLETTRTVRKPLMEKKKKQRAERKEVQVGQKPKTSVQSSSKVRKSILMSKRN

>DcSOFL1

MDTSNSQIFGGSQEECHSSESGWTMYIGSPVNEDNDDGDQVENDYVEDEYKGGGDVESDDSMVSDASSAMAANMHNMYQKQVKEDDEDVDLKQISRKVVKKVEKNANQGKKKDMESAGMNVAPAQSGNKFSGARSWTLGTTTYAGEICFAVIIAVLGLLLFALLIGNIKYACKFFMKFNG

>DcSOFL2

MDTSNSQIFGGSQEECHSSESGWTMYIGSPVNEDNDDGDQVENDYVEDEYKGGGDVESDDSMVSDASSAMAANMHNMYQKQVKEDDEDVDLKQISRKVVKKVEKNANQGKKKDMESAGMNVAPAQSGNKVRRNWLGRKKWTHLFDFIIFSRFIIFLFNYFI

>DcSOFL3

MDSYHSQFFGGYEEECQSSESGWTMYIGSPADDDDDGEAKNDVEEQYKGGGGDVESDDSMLSDASSAMTSNIKKKDVYHQEAKKGDVELKKNARKSMKKGAEKIEKPEKKKDEKSTSMNAAPQSGNKVRRNWLGKKK

>DcSOFL4

MDSYHSQFFGGYEEECQSSESGWTMYIGSPADDDDDGEAKNDVEEQYKGGGGDVESDDSMLSDASSAMTSNIKKKDVYHQEAKKGDVELKKNARKSMKKGAEKIEKPEKKKDEKSTSMNAAPQSGNKGIIVVVLIDFPEQIEENGSKFSHVISWVVDYVI

>DhSOFL1

MDSSKVHQETEECSSNESGWTMYIASPDQELDYDDYEDEEEYIIGEQTYTKDPDKHTEEVDSDDSMASDASSGPSYQGILYERVDRIHGSGHAANETGSKLVDYKKHQQPERKKLDEQKKEAKDKNGNKKRNAATSKNK

>DhSOFL2

MDSSKILEDTEECSSNESGWTMYIASPVHELGYDDYEDEEYCSTDEQIYERDGAGREDGDVDSDDSMASDASSGPSYQGQSYGFRGFGHAWNKSQNKHVDEKNHKQEEEKHFKQKKAAKDKFECDKISVKKLKNK

>DzSOFL1

MEASHNTRGLEDEQHGSSESGWTMYIGSSIHENDHYDFIGEYSYNTSKQVEGYNKNRCPKYDGNGHNDDESDDSMASDASSGPILHQLPSSSDQNLGMDYYKHEPFKSFSAEKLHKQVIKRDQRRNKFGKEKLELKALSAASHAQSRDKVKITNNLRLKE

>DzSOFL2

MEPSKIFGGKEECHSSESGWTMYIGSPIQGGDDDDDDNGHSEADDDAYAGGHADETYANHEADSDDSMASDASSGPSHQGVQFHGNMEGGHGTSYFKHDEDEEEGNCYSDKKAKKSVEKQKLGMKKKENKEEKEQMTFNAKGATTSRSGSKVRKSMWLGKRK

>EgrSOFL1

MEYSPNHSGAEGFSSSESGWTMYIASPTKEDDSGCTEASWVTQKDQITTISPPGNRRKEGSDDSDDSMASDASSGPSHHRQKHGKDGCSKPDKKQEWFSNEKSKRKEKKKEKGSVVKGPKR

>EgrSOFL2

MESSSQAFEGSEECLSSESGWTMYIGSPMQEEDEGAGDEQSTHNDADDAEDGGGKVGRDGEAESDDSMASDASSRPSYRGRVASKNGKSCAAKKGQSRNAYKEEEEEDDDDDDPKQRAYGTNPSPSKGKVSKSLRMKKKK

>EgrSOFL3

MRWAMESSSQAFEGSEECLSSESGWTMYIGSPMQEEDEGAGDEQSTHNDADDAEDGGGKVGRDGEAESDDSMASDASSRPSYRGRVASKNGKSCAAKKGQSRNAYKEEEEEDDDDDDPKQRAYGTNPSPSKGKVSKSLRMKKKK

>EguSOFL1

MDSGCSSSESGWTMYIASSPETQCSSSSSSEYYYDEGGAGKKGGGDDVESDDSMTSDASSGRCRPSDYHGLGQVLNKNERKLHESSKKKVQTKKLHDGGGEKKKKKKMKLQHGNNKRIDDDDDDDGDDAASSEDRKPPPPDIYSAGRRWNKR

>EguiSOFL1

MESSSLIREAEECSSCESGWTMYLSSPMHDGGDSDTEVKSTDEEEDHDNDHNSNKSNGSNEDDEDNDSMASDASTGPIQHRHGDTKCDRSSVMGLPNNDDDDNELENDERNQQSSYSYNKVCGVNKMRNGGRIAVSHREDDAALFHTSSKMRNLTINTDDN

>EguiSOFL2

MESSPLIMEAEECSSSESGWTMYLASPMHDGGDGDIEVQANDEEADDANDHNSNESDDGSEGEDNDSMASDASSGPIQHKHGDAKCDQSSLTYHLDHDDDEHGLEEDESNQYYLSYSCKKFCELNKMRSGRRVGVSEKEDSATSLFNTSSKVRKASGK

>EguiSOFL3

MEASQPTGDAEECSSSESGWTMYLASPMHDNGDSDDHDDLEEDDARGEDDDDDGDGGGSNNNSSVGKDEGDEEDDDSMASDASTGRAHNKYSYRKSDGIKALDHPKPDEESDGDYSQPSSHLCRIFPKLKKNRDGKTRNPFQREAGSTSSQNNPKGSKPNLSHK

>EguiSOFL4

MESSSLIREAEECSSCESGWTMYLSSPMHDGGDSDTEVKSTDEEEDHDNDHNSNKSNGSNEDDEDNDSMASDASTGPIQHRHGDTKCDRSSVMGLPNNDDDDNELENDERNQQSSYSYNKVCGVNKMRNGGRIAVSHREDDAALFHTSSKMTTSSKRS

>EguiSOFL5

MESSSLIREAEECSSCESGWTMYLSSPMHDGGDSDTEVKSTDEEEDHDNDHNSNKSNGSNEDDEDNDSMASDASTGPIQHRHGDTKCDRSSVMGLPNNDDDDNELENDERNQQSSYSYNKVCGVNKMRNGGRIAVSHREDDAALFHTSSKVRKTFEK

>EsSOFL1

MEREECCSSESGWTTYISSPMEEDEEEVIDEVYYEGHDIDKDRRKYANEYENNKDSDDSMASDASSGPSYPQTSNRGRRREGIILRNRESENNSKSNGVYDHKHDDKNSGNHKCRTKEKKKGDYKSRSYKKK

>EsSOFL2

MESPRIHGGTEEKTSCESGWTMYIEDTFHGNHHRSDVVYQDYDEDGDDGDFRVKDDGEDGGDDDDDDDDSSDNRSDDSMTSDASSWPSTQLPRNTKNHAAAAKNSNAKQVNHQTKNRACEKFSDQEEESELKARTRTTANSRVQSRGKVSKTK

>FvSOFL1

MESSQVVGAEEWTGSGESGWTIYIASSIHGERDGVYSSSHNTTKGRKVIFEHEDDGEESDDSMASDASSGPSHPERSRGATRLRHAEKEGAKKHSTEKKSKNKHETAMIKGEKAVEDILQHKADSAASHI

>GaSOFL1

MEFSKVVVEDCSSSESGWTAYIASPEEEEQQQKNLLSFTEDGSSSISVKHFKKNEAAEDADSDDSMASDASSAPSDQRQHFFVHARDEREKCVPGKNNTQQDENKKKKKKNNKQIVKGAEGKDQQSVMQSGGGRRSNNI

>GarSOFL1

MEPSNIFGGAEECHSSESGWTMYIGDAAADDDGDAGTDADHDDDDDDAETDDSMASDASSGPSHRLEQEVEGEEEGRHCYSDKKARKSSVGSKQKPGTKRKEDKEEMRRLKTKESSTQSPSGSRKNIWFGKRK

>GarSOFL2

MEASHILTSFETEEQSSSESGWTMYIGSSIHENDYNTYEQARHHKNHQCPNGYNEDESDDSMASDASSGPSHHKFPPSSEQNLGMNHFTHGSIVKSISTEKLQKQVIKRDQRRNRHGKGKLKLKTISAPSRVQRGYISKDDEAGRVGVSQISQSCLEMCDEC

>GarSOFL3

MESSEIYGGIEECHSSESGWTMYIGSPIHGGDDSGDGHSEKADDEGVYGVDNHADEDADSDDSMASDASSGPSHKGHRGTTTLYFKHDEEEEDDDKDERNFFSGKKDRKSKEKKHKVGLMKNKQVKKQTPLKTKTSFI

>GhSOFL1

MEASHILTSFETEEQSSSESGWTMYIGSSIHENDYNTYEQARHHKNHQCPNGYNEDESDDSMASDASSGPSHHKFPPSSEQNLGMNHFTHGSIVKSISTEKLQKQVIKRDQRRNRHGKGKLKLKTISAPSRVQRGYISKDDEAGRIGVSQISQSCLEMCDEC

>GhSOFL2

MEASHILTSFETEEQSSRESGWTMYIGSSIHENDYNTFEQESHHKNHQCPNGYNEDESDDSMASDASSGPSHHKFPPSGEQNSGMNHFTHGSIVKSISTEKLQKQVIKRDQRRNRHGKEKLKLKTISAPSRVQRGYISKDDEAGRLGVSQISQSYLEMCDEC

>GhSOFL3

MESSKIYGGIEECHSSESGWTMYIGSPIHGGDDSGDGQSEKADDEGIYGVDNHADEQADSDDSMASDASSGPSHKGHRGTTTLYFKHDEEEEEEEEDEDERNFFSGKKDRKSKEKKHKVGLMKNKQNKQVPLKTKTSFI

>GmSOFL1

MEPPMLGVEEECHSNESGWTMYIGSPRDEDAQHCDDDDDNHSLEEDYYDYEAAAAQHCDVESDDSMASDASSGPSKSKEQVKKNNKYCSEKKVEKKNEIMAFANSMDKAPAAVQNGSKVKVNKNYRVGTRKL

>GmSOFL2

MDSFKQIWGSAEGCSSSESGWTMYIASPMQEDDAGCSNENDGYHDNNIYGENRRKKQGAQVDEEESDDSMASDASSGPVHYHHAYTHRQSSHGTAASKKDKQDHGSKFSSKKNANKQEKKRVDSRSKK

>GmSOFL3

MDSFKQIWGSAEGCSSSESGWTMYIASPMQEDAAGCSNENDGYHDNIYAENRRKKQGAKVDEEESDDSMASDASSGPIHYHHAYAHSQGSHGTAASKKDKQDHGSKCSSKKNANKQEKKRVDSRSKK

>GmSOFL4

MEPFGAEGCHSSESGWTMYIGSPIDDAGHSSDNDDNNKKGTQAHPQDDDDDDDESDDSMASDASSGPSHHHGFADFRRDAEEENDENKYCLEKKAGKTQHKQMEGKKVEKNGMLIVDSKDKSPVQGCGKVRKNYFVGKRK

>GmSOFL5

MEPFGAEECHSSESGWTMYIGSSIDDAGGHSTDDGDGDDDNDDDDKEEGTQAHPQDDDESDDSMASDASSGPSHHHGFADFRRDSEEGNHDKYCFEKKASKTQHKQMEGEKVEKKGMLPVDSKDKSPVQGCGKVRKNYFVGKRK

>GmSOFL6

MEPPMLGVEEECHSNESGWTMYIGSPRDEDAQHCDDDDDNHSLEEDYYDYEAAAAQHCDVESDDSMASDASSGPSKSKEQVKKNNKYCSEKKVEKKNEIMAFANSMDKAPAAVQNGSKVKGIACELEGTPSN

>GmSOFL7

MEPPMLGGEEECHSNESGWTMYIGSPRDEDAQHCDDDDHHHHSFLEEDYDYESAAAAAQHCDVESDDSMASDASSGPSKSKEQVNKKDNKYYCSEKKVEKKNEIMAFNSKEKAPAAVHQNDSKVKVNKNYMVGTRKS

>GrSOFL1

MESSKIYGGIEECHSSESGWTMYIGSPIHGGDDSGDGHSEKADDEGVYGVDNHADEQADSDDSMASDASSGPSHKGHRGTTTLYFKHDEEEEEEEEDEDERNFFSGKKDRKSKEKKHKVGLMKNKQKKQVPLKTKTSFI

>GrSOFL2

MEPSNIFGGAEECHSSESGWTMYIGDAAADDDGDAGTDADDDDDDDDGDRFDSADESQTDANHEAETDDSMASDASSGPSHRLEQEVEGEEEGRHCYSDKKARKSSVGSKQKPETKKKQDKEEMRRLKTKESSTQSPSGSRKNIWFGKRK

>GrSOFL3

MEASHILTSFETEEQSSRESGWTMYIGSSIHENDYNTYEQESHHKNRQCPNGYNEDESDDSMASDASSGPSHHKFPPSGEQNSGMNHFTHGSIVKSISTEKLQKQVIKRDQRRNRHGKGKLKLKTISAPSRVQRGYISKDDEAGRIGVSQISQSYLEMCDEC

>GsSOFL1

MEPPMLGVEEECHSNESGWTMYIGSPRDEDAQHCDDDDDNHSLEEDYYDYEAAAAQHCDVESDDSMASDASSGPSKSKEQVKKNNKYCSEKKVEKKNEIMAFANSMDKAPAAVQNGSKVKVNKNYRVGTRKL

>GsSOFL2

MDSFKQIWGSAEGCSSSESGWTMYIASPMQEDDAGCSNENDGYHDNNIYGENRRKKQGAQVDEEESDDSMASDASSGPVHYHHAYTHRQSSHGTAASKKDKQDHGSKFSSKKNANKQEKKRVDSRSKK

>GsSOFL3

MDPSQDLASEVEFNGTESGWTTYIGSPIYNEQSNGDDEHSVDFEDYGNNYKNSHRSIYDDDNKRENNDDFNRDTDEESDDSMASDAASGPSHLHLVRINSEGSHGGLDFTEHTENDNDKIRLTKRTTKQVRKTRYEGIVGEKEEASLLVADSAASHA

>GsSOFL4

MEPPMLGGEEECHSNESGWTMYIGSPRDEDAQHCDDDDHHHHSFLEEDYDYESAAAAAQHCDVESDDSMASDASSGPSKSKEQVNKKDNKYYCSEKKVEKKNEIMAFNSKEKAPAAVHQNDSKVKVNKNYMVGTRKS

>GsSOFL5

MDRSKDLASEVEFNGTESGWTTYIGSPTYNEVSNGDDEHSVYFEDYGNNYKNAHRNIYDDDNKRENNDDFKRDTDEESDDSMASDAASGPSHLHLVCINSEGSHGLDFTEHTENDNEKIPLTKRASKQVRKTRYEGIVVEKEEASLFVADSAASHV

>HaSOFL1

MEFSKYLGVADDECSSSESGWTMYIVSSMHDETTNHHQHDHVHVYDHDDNTHGGYYKMHHNEDSDDSMVSDASSAPSRQRGISKKRSGLRRESKHALNQKQEEKKLYDRRQRAVKEEHKTRIDAKTKKT

>HbSOFL1

MESSGILGFIEECSGTSSKESGWTTYIASPIQENHHHHDDDDHSNDKQADCKKGNYNKVDDGTESDDSMASDASSGPSHHELPSKINEISVDTGPSKHAITKYSSKEKLGKQFKQSEGERPRIKLGREIGSAASDGHGGAKVRKTK

>HbSOFL2

MESSKLFGGAEECHSSESGWTMYLGSHIHGDDDDDDLHSYDDDDDDDDDDGGNGKNYYHEDDDSDDSMASDASSGPSHQGTDFKLQKDADGKYQSQRKPNNKQVEKQQQKAERRRKEENKDQEVGFMARRAYNKSAPAQSSSKVRKSISWMGKEK

>HbSOFL3

MESSQLFGGPEECNSSESGWTMYLGSPIDGDDDDDQHGNKDEDDEDDDDDGDGGNGKNYHHEDHSDDSMASDASSGPSHQGTAHDKPQKDFDGKHLPEKKHNKKEAENQQKTERRRKKIKKWDLWQEDHNKNASAQSTSKARESISWMGKR

>HiSOFL1

MDSSKYLQGNEECSSSESGWTTYIASPDNETDQSEYEDAMTVEQVGGYKKDDVEEGVDSDDSMASDASSGPSDQRHVYRNESKYVVGKKHIQQEEKKKQQHQQIKAEKDKPGQSAGRSKKM

>HuSOFL1

MEAPHILGGMEDEEHSSSESGWTMYIGSSTQENDHYNYIDEYDCDTHEQEEDYHKNQRGNYDGNSQNDDESDDSMASDASSGPSHHKLPCSSEQNLGMDRHKLEPLKSTSTEKLHKQVIKRDQRRNKIEKEKLEGKATSAASHVRSGEKVKIINFMSQEE

>InSOFL1

MESSHVYGAPEECHSNESGWTMYIGSPTTDEDDDHLNTNNVNGDSYEAEEENSDDSMASDASSGPSHWWVNGKIHSKNDKDSIVMMRGKNHGYSDKSFPVPNCKTSKNPSSSVKKKKEKNGGGGGEKEESSVHSAKSHKERRSCGGGFWKGKTGK

>JcSOFL1

MDSSKHHLGTEGCSSSESGWTMYIASPMEEDDNECSDHVVDDNDHNTIAIDGNFNDEGGDEQDSDDSMASDASSGPNHRYKYENGQTSRGTTSVKQDKGNNFNHCSPTKPNKKEKSYENSTEKNRRFPANRKYSK

>JcSOFL2

MGSSQFFGGKEECHSSESGWTMYLGSWVQDDGDDDDGDNNDNDHHGGNYGKNKNNYYQEDDSDDSMASDASSGPSHQVKNEDEDDDDDHNGKYHSERKNNKEKQKTERIRKKNGKEAEVESMARKADNNNYFPAQSCSKQKML

>JcSOFL3

MNAFAQECSSGCESGWTLYLEQSFLGNNKKQRNNNSTRSCENYVKDEEIMMREHNDRDDNHDDEEEEDLSMVSDASSGPPPHQNVHEEESYSFNYYDNGGFYPAFNDTTLINNGGIKRQSEHRRRGGDKEQEIVPANFLDDTASSPAINWSKNQASMQSTLDYSQAAAGFSATHFEGISAYQDHFGYIQPSSLSGNQLQNNQWF

>LaSOFL1

MEPYNIFGSGEECHSSESGWTMYIGSPTYDDDGEDIDNGDDEGIIPVNDLKEDHESDDSMASDASSRPKGNNGLSHFLQVAEEEKLEKKEKKIQEKHIEGKREEKKERVTIDGKGKVPVKCNSKVRKNYLVEKMK

>LaSOFL2

MEPSRDLASAREGDFNGSESGWTSYIGSCIYSEVYIDDEKNVHMEDYNYKNANGKVIVVHDDDKYDERAENNNKGACDEESDDDSMASDASSGPSHFQLVCINNEGRSNGLVYKKQKENDNEKIFSSKRGSKQVRKTKYEYKVEKEEEDSVLLIADSAASHV

>LaSOFL3

MDPSRDLASEGEFNGSESGWTTYIGSHNYNEDEQSVDVDEYGYNYKNGHGKVDNYDDNKGTGDEESDDDSMASDASSGPSHFQLVCINSERKHTENDNEKILSAKQTNKEVTMKTRYEGMVEKEEEDSLQLIADSVCSHA

>LaSOFL4

EGCSSSESGWTMYIDSPVQDDDAGCSNEYDGYREVYGENRRKKQGNKVDEESDDSMASDASSGPIQYQNSYARGESSQGIAVSKKDKQDHGSKCSSRKNNANKQEKKGVDSRSKKR

>LaSOFL5

MDSYKQIWGAEGCSSSESGWTMYIDSPVQDDDAGCSNEYDGYREVYGENRRKKQGNKVDEESDDSMASDASSGPIQYQNSYARGESSQGIAVSKKDKQDHGSKCSSRKNNANKQEKKGVDSRSKKR

>MaSOFL1

MNMESSSEVTAEGEECDSSQSGWTMYLATPMHDGGQVVADGEHDDDDSLASDASSGSVTHGSEVTDQLEVDEEETCSQYPSRE

>MaSOFL2

MNASQLPGDVEECSSCESGWTKYLSSPAHDDDGSEVESVVGDAGEDTGHESKESDDDSMASDASTGLIQSKDSDGMVHSKCNDDDDDDDDDDDGFRNKKQTQCSSHSNKQFCKVEEARSEREDFAALHSNSKARKKKLK

>MaSOFL3

MEPSTVGVLSSSSVAVVMNTSQLTGDDAEELSSCDSGWTKYLSSPTHHDGGDDDDDDDDSEVEFTGGDDDDDDEHKGYDSEDSDDDSMASDASTGFIGSGVDGSKCNDVGDNMDGNGKEGHAQCSSSYSHEEFYEVEEARSKQACDPRSNSEARKNRFK

>MaSOFL4

MSSEYSSGCQSGWTLYLAHSLDDQNSLYHHDGSSFRGEEEEDLSMVSDASSGPPLLHGDDDHGCCYHNSDTCLHTAAPPSAALGGDGAKKKRAVAPQRKEYSAALDDTASSQLFSSSEACCSFCSLSLPAFAYR

>MaSOFL5

MQREEADVSSDCSSGCQSGWTDYLDKSCGECPQPLVCGKGGSFEEEEEDLSMVSDASSGPPHFPEEDEHSCGYLRSSTCFHAGGCLCSALTPAAGLAAGGAKKRRVEPEQQRQRSSLLDDTASSPLFSFSKACYIPDPYSPSSDFNPLLSFNEAISEFSCGFKRNLQLEKQMGHLQSSVPETS

>MaSOFL6

MMLGEEEDVSSECSSGCQSGWTAYLDQSSYDSPQPLVYNKAGYLQEEEEEEEEEDLSMVSDASSGPPHFHEEDEPSFCYLHSSTCFEGGGCLCSALTPAAGSAKSGAKRKRVEPEQQREHSSLLDDTASSTLLSYPKTRFNGDSNSNNYLKPPMEGVLEFSCGFSATHFKRNHELHKQVGYLQSSSPVKPTPTPVLTKEGGKKIW

>MdSOFL1

MEPLRNFYGTEGCSSSESGWTTYIASPMQEDEPECSNIEDVGYENHHHIAYLTRKKVGKDDNESDDSMASDASSGPSHHHDLVPSRSKDSKGTARSKRDNNKQSRHKSTSKPEKKTGEKSTKRK

>MdSOFL2

MDPYRNFYGTEGCSSSESGWTTYIDSPMQEDDAEYSNIEDVGYKNHHYIAYLTRKKVGKDDNDNESDDSMASDASSGPSHHHDLARPRSKETKGTARFKRDNSKQSRHKSTSKPEKKTGERNTKKK

>MdSOFL3

MESFQVXGGDAKKWSGSSESGWTMYIGSPTMRSESNEVYYISDXKKGGGKINGKNCIVDRHDGESDDSMVSDASSGPSHREEVLLPRGIGGRNRSGGWRLRHADRDKKVTCRKEKKREDERLRVRXKGGRRRXCCTRQIVLKVRCEVWTAVEQPIKVIKKD

>MdSOFL4

MESGKMLGGAEECHSSESGWTMYIGSRLEGENDGGMHHEEDDEGGENYGVHRKGEESDDSMVSDASSGPSHHHGRPSRSGQGVAAAEKKGKKKPAKEKQTRGGRRKQEEKKEKALLIHGKKR

>MdSOFL5

MESFQVVGGDAKKWSGSSESGWTMYIGSPTMRSESNEVYYNISDRKKGGGKINGKNCIVDRHDGESDDSMVSDASSGPSHREEVLLPRGIGGRNRSGGWRLRHADRDKKVTCRKEKKREDERLRVREKGEEEEELLHKADSAESQV

>MdSOFL6

MESGKVFGGVEECHSSESGWTMYIGSHFEGENDGGMHHEEEDDDGGTKYGGYHKDEESDDSMVSDASSGPSHHHGRPCGKERVAAEKKGKKKPEKEKQTRGGRRKQEEKKDKALLIHGKKR

>MdSOFL7

MESGKMLGGAEECHSSESGWTMYIGSRLEGENDGGMHHEEDDEGGENYGXHRKGEESDDSMVSDASSGPSHHHGRPSRSGQGVAAAEKKGKKKPAKEKQTRGGRRKQEEKKEKALLIHGKKR

>MdSOFL8

MNISPSQYNSGCESGWTSYLDQSSYLSQSQFQKPGGFADYVETKEEEEEEEEEEDLSMVSDASSGPPHYHDFDEDCVYEDGSSFSVSWGSKLGKKSKKKSKGNGSRELHLDDTASSPVLNYSKKKLSTNFSKDESSMQNVLGYSEGFSATHKKGKSALLQHFGFLKSSLPKSPASQKPVIKQVICKVKVGSDIYNDRNEVSHFICCSWGEKRSGNKQCKS

>MdSOFL9

MNISPSQYNSGCESGWTSYLDQSSYLSQSQFQKPGGFADYVETKEEEEEEEEEEDLSMVSDASSGPPHYHDFDEDCVYEDGSSFSVSWGSKLGKKSKKKSKGNGSRELHLDDTASSPVLNYSKNVLGYSEGFSATHKKGKSALLQHFGFLKSSLPKSPASQKPVIKQVICKVKVGSDIYNDRNEVSHFICCSWGEKRSGNKQCKS

>MdSOFL10

MNISPSQYNSGCESGWTSYLDQSSYLSQSQFQKPGGFADYVETKEEEEEEEEEEDLSMVSDASSGPPHYHDFDEDCVYEDGSSFSVSWGSKLGKKSKKKSKGNGSRELHLDDTASSPVLNYSKKKLSTNFSKDESSMQNVLGYSEGFSATHKKGKSALLQHFGFLKSSLPKSPASQKPGDLQGESWE

>MdSOFL11

MNISPSQCNSGCESGWTSYLDESSYLSQSQFQKPGGFADYVETKEEEEEDLSMVSDASSGPPHYHEFDEDCVYENRSSFSVSWGSKLGKKSKKKSKGXGSREQHLDDTASSPVLNYSKKKLSVNFPKDESSMQNVLDYSEGFSATNIKGKSVLLQHFGFFKSSLAKSPASQKPGDFQGGSWE

>MdSOFL12

MNISPSQCNSGCESGWTSYLDESSYLSQSQFQKPGGFADYVETKEEEEEDLSMVSDASSGPPHYHEFDEDCVYENRSSFSVSWGSKLGKKSKKKSKGXGSREQHLDDTASSPVLNYSKKKLSVNFPKDESSMQNVLDYSEGFSATNIKHFGFFKSSLAKSPASQKPGDFQGGSWE

>MeSOFL1

MESSQLFGGAEECHSSESGWTMYLGSHIHGDDDDNQHSYEKDDDDDDDYDGGNGKNYYHEDDSDDSMASDASSGPSNQGTDFKLQKDVNGKYYSQRKPNNKQVEKQLQKSERRRKEDKNKDEEVRANNKTTPTQSTSK

>MeSOFL2

MESSKLFGGPEECHSSESGWTMYLGSPIHGGDDDDENDDKHSNKDDDDEDENGGNDKNHYIEDDSDDSMTSDASSGPNNQGSAHYKLQKHVDDNKHLPEKKQENQQKAERGMIKKWDLLQEDHHNKNAPAQSSSKVRKSISLIGKRN

>MeSOFL3

MESSQLFGGAEECHSSESGWTMYLGSHIHGDDDDNQHSYEKDDDDDDDYDGGNGKNYYHEDDSDDSMASDASSGPSNQGTDFKLQKDVNGKYYSQRKPNNKQVEKQLQKSERRRKEDKNKDEEVRANNKTTPTQSTSKVRKSISSWMGKRK

>MeSOFL4

MESSRILGRTEGCSGNSSNESGWTTYIASPIRENHHHLDDDDDDDHSTDNQADYRKGTYNKVDDGTESDDSMASDASSGPSHLEFPCKINDISVGIGPSKHAITKYSSREKLGRQFKQNEGARPRTKPGKEVESAASDVHGGATVRKAK

>MeSOFL5

MDSCKLNLGTEGCSSSESGWTLYIGSPMQEDDDECSDKDNDNDQNTIANDGNAGDDYDDEQYSDDSLASDASSGPNHRHKPEGGLQSNHGTASFKHENGNNFNHCAPATKSNKKEKKNDGDSRVKDRRLSDNRKYSK

>MnSOFL1

MSTEGCSSSESGWTMYIASPMQEDDADCSSDDDNDKKKRLAYHRYAENDEDDTNSDDSMASDASSGPAHHHNVRSRGKDRQDASRLKHDKKKHSSHNNTSKKERRNREKSPRRK

>MnSOFL2

MESSQVLGDSDQKCSDSESGWTMYIGSQICKENYGEADDDGDEKDNENKPISHEGGSCGGDESDDSMASDASSGPILHQLTPDGIKQGTEMFGHLGNTVGKCFSANIKPCKQEKKRDERRIKQEVPKADSAESQV

>MnSOFL3

MESSQVFGGAEECQSSESGWTMYLGSPQNDDENDQQISDYYGENNDDDSNSSNNNDDDNKGGKRHDNDDGDDDDSMASDASSGPKNHRIERRRFDGESSTFYQREEEEDDDDDGGDREDGKKCCKSLDKEGKKSALSTRKKKQKQRAERRKEK

>MtSOFL1

MDTLASECSSGCESGWTLYLDRSLNTSQSHTPSLGCYEETKHKKAQNIEDSDEEDLSMVSDASSGPPHHDSYFNAQDCSASKPTKLVAKRSKKRQKVQENNNIQQHLDDTASSPLFDNNNVVTMSNQQTTSTTESMLDYSQGFSATYFEERSSLQDHFGFLQPSLSQNEAHINKKWYGGEEMGMI

>MtSOFL2

MDSFKQIWGAAEGCSSSESGWTMYIDSPMQEDDARCSNENDGYHRHEIYGEKNRRKKKVDEEESDDSMASDASSGPMNYQHQTNYGRGQGKSGLKKDKQDHGSKSCSKKNGNKQEKKRVDSRSKK

>NaSOFL1

MEYPKKNGGITEECSSNESGWTMYIGSPYRDGEDDDDDEGTPRKGYKNLDYIEEGGGSDDSMTSDASSGPSQQGVSTSVEQRYGNHEFKHAEKDNRKFTSKEQQRQAKKKLIDKNVKAAKEDPGHKAKSGKNYGYCRSTTRGKHVN

>NaSOFL2

MSKEEECSSNESGWTMYIGSPYRDGEDDDDDEGTPRKGYKNLDYIEEGGGSDDSMTSDASSGPSQQGVSTSVEQRYGNHEFKHAEKDNRKFTSKEQQRQAKKKLIDKNVKAAKEDPGHKAKSGKNYGYCRSTTRGKHVN

>NnSOFL1

MESSQLYGGTEEYCSSSESGWTMYIASPMQNDDDDHGYDYEEEDDDDDDDDDDDDDDGNNGNDDSHDDDNDSGDSMVSDASTGPSHREHPYEHGDERAHGMTQFEHNQDEDDNEYCSYETPYRSMKKREQRGISKEKEDSVLMANSATSHVQNGAKVRKTNWMGKRN

>NnSOFL2

MESSQLFGGTEECSSSESGWTMYIASPIHNDDGDDDHHDYEEEDDDGNDGNSSDDDNDSDDSMASDASTGPSHREHPFEHGEDGHGVGRLEHDQDEADDEYYPHKSSHRNMKKGGERRGTRKEKEDSVPANSATSHVQNGAKVRKTTWFKKGK

>NtSOFL1

MSKEEICSSNESGWTMYIGSPYRDYNHGEDDDDDEVTPRRGYKNLDYIEEGGGSDDSMTSDASSGPSQQGVSTSVEQRYGNHEFKHAEKDNKKFTSKEQQRQAKKKLIDKNVKAAKEDPGHKAKSGKNYGYCRSTTRGKHVN

>NtoSOFL1

MKSSKLSGGTEECSNESGWTMYIASPIHQYNQEDDDDGDERSSRKRDKYLYNDDDDNEDGESDDSLVSDASSGPGHQEVCTSIGRSHGKLQFKHAEKETKKFSSKEHRGQVKKKLYDTNIKAAKEDSGHKAKNKIDYACSRSTSRRKY

>NtoSOFL2

MSKEEECSSNESGWTMYIGSPYRDYNHVEDDDDDDDEGTPRKGYKNLDYIEEGGGSDDSMTSDASSGPSQQGVSTSVEQSYRKHEFKHAEEDNRKFTSKEQQRQAKKKISDKNVKAAKEDPGHKAKSGKSYGYCSSTREKHGN

>NtoSOFL3

MKPASQNFNEEEECHSSESGWTMYIGSPSNSTEDDKLNVADFEEEDDVGDNKNERNQEEDDDTDDSMASDASSGPSHMREHFVRNAKSTTGARYVNPKEKGNGKGCSNNKANTKNGIKNQDKEESVFSAKGAKVPSNGGKVRKSIWKGKGK

>NtoSOFL4

MDSSKLFGEAEECNSSESGWTMYIGSPSNGEVEDELENEDFDELEDNNKEAINSDDKEDGDTDDSMDSDASSGPSHRQYITKNGKSAGLANMVHYKNPKEKGKDHKVCSLNNKANNSMKSGYKNEVKEEESVFTAKGAPSNGGNK

>NsSOFL1

MLLTEECNSSESGWTMYIGSPSNGEDDDELENEDFDELEDNNKEVINRDDNEDGDTDDSMASDASSGPSLRQYITKNGKGAGLANMVHYKNPKEKGKDYKVCGLNNNDPMKFHKANNSVKSGSKNEVKEEESVFAAKGEPSNGGNKVRKSIWMGKGK

>NsSOFL2

MEYPKQLXGITEICSSNESGWTMYIGSPYRDYNHGEDDDDDEVTPRKGYKNLDYIEEGGGSDDSMTSDASSGPSQQGVSTSVEQRYGNHEFKHAEKDNKKFTSKEQQRQAKKKLIDKNVKAAKEDPGHKAKSGKNYGYCRSTTRGKHVN

>NsSOFL3

MESSKLTGGTEECSNESGWTMYIASPIHQYNQEDYDNGDERNSRKRDKYLYNDNDDDDNEDGESDDSLVSDATSGPGHQEVCTSIDRSHGKLQFKHAENETTKLSNKEHHGQVKKKLYDTNIKAAKEDSGHKAKSRIGYACSRSTSRRKY

>ObSOFL1

MESPQLTEDDGEECNSNESGWTMYLASPTRSDDVRAIVSEGSNVDDGTGYSNVNHRGEDDKCNANDDADYDSLASDASTGPAEVKVQEGKEDRDHQMNGGSRHEHGKETQDEIRPKLSISCNKKVGKMKKGEEKISRRGQNKRHEIYCLEDINIPSYHTSI

>ObSOFL2

MESSHITGDDGEGCNSSESGWTMYLASPMHGDDDGGGKVSGSEGSNVDDGYGYISGRGSRKKYEDDGDGDDDDSLASDASTGPAKVKVPSTPDGDEDGGRRKHHDDDDGGGKEEEEEEDHGMHTKFLISSGKKAGKTEKGGEGKSSKKGHNKRGSSSRTRFFW

>OeSOFL1

MDSSKILEGNEECSSNESGWTMYIASPIDKYNLDDEDDHSGSIDKQEDGLPAKDDGDTDSNDSMASDASSGPSHREHLRNHVRGRHCPGVFGHAGDKDNRKRLGKKHHQQEEKQPCDEQIKAAKDKPGHKGNSAGRLKKK

>OsSOFL1

MESSHITGDDGEGCNSSESGWTMYLASPMHGDDDGGGKRSGSEGSNVDDGYGYISGRGSRKEYEDDGDGDDDDSLASDASTGPAKVKVPSAPDGDDAGGRRKHDGDEDGGGKGEEEEEEDHGLHTKFSMSSGKKAGKTEKGGEGKSSKKGQNKRGSSSRTRFFW

>OsSOFL2

MESPRLIEDDGEECNSNESGWTMYLASPTHSDDVRAIVSEGSNVEDGSGFSNERRRGKENKGHANDDGDYDSLASDASTGPAEVKMQEGKEEKDHQMNGGNRHQHAKDEQDEIPTKLSTSYSKKVGKIKKGDEKTSRRGQNKRHEIYCLKELNLPYFHIRIKQCT

>OsSOFL3

MESPRLIEDDGEECNSNESGWTMYLASPTHSDDVRAIVSEGSNVEDGSGFSNERRRGKENKGHANDDGDYDSLASDASTGPAEVKMQEGKEEKDHQMNGGNRHQHAKDEQDEIPTKLSTSYSKKVGKIKKGDEKTSRRGQNKRRSSSRTSFFW

>OtSOFL1

MESSQLSGDDGDKECNSNESGWTMYLASPRSSDDVKTNDSGESNVEDGSAYSNGRSKEDYTNYGDGDYDSLASDASTGPAQVKTP

>PbSOFL1

MDPYRNFHGTEGCSSSESGWTTYIDSPTQEDDAECSNIEDVGYKNHHYIAYLTRKKVGKDDNESDDSMASDASSGPSHHHDLARPRSKETKGTARFKRDNSKQSRHKSTSKPEKKTGERSTKKK

>PbSOFL2

MESGKMLGGAEECHSSESGWTMYIGSRLEGENDGGMHHEEDDEGGENYGGHRKGEESDDSMVSDASSGPSHHHGRPSRSGQGVAAAEKKGKKKPAKEKQTRGGRRKQEEKKDKALLIHGKKR

>PbSOFL3

MESGKVFGGVEECHSSESGWTMYIGSHFEGENDCGMHQEEDDDGGKNYGGHHKDEESDDSMVSDASSGPSHHHGRPCGKERVAAEKKGKKKPEKEKQTRGGRRKKEEKKEKALLIHGKKR

>PbSOFL4

MEPFRNFYGTEGCSSSESGWTTYIGSPMQEDEPECSNIEDVGYKNHHHIAYLTRKKVGKDDNESDDSMASDASSGPSHHHDLVPSDSKDSKGTARSKRDNNKQSRHKSTSKPEKKTGEKSTKRK

>PbSOFL5

MESSQVAGGDTKNWGGSSESGWTMYIGSPIMRSESNEVYYNISDHKKGGGKINGRNCIVDRHYDGESDDSMVSDASSGPSHREEVLLPRGIGGRNRSGGWRLRHADRDKKVTCRKEKKREDERLRVREKGEEEEEELLHKADSAESQV

>PdSOFL1

MESSPLIREAEECSSCESGWTMYLASPMHDGGDSDIEVVSNDEEEDHDSDRNSNKSNGGNEDDEDNDSMASDASSGPIQHRHGDTKCDRSSVMGLPNNDDDDNELEDDERNQHSSYSSNKVCGVNKMRKGGRIGVSHREDGGTLFHTSSKVRKTTEK

>PdSOFL2

MEAEECSSCESGWTMYLSSPMHDGGDSDAELQPNDEEEDDANDRCRNESDDGSEGEDNDSMASDASTGPIQRKHGDTKCDQSSLICRLEHDDDDDGIEEDERNQYHLSYSCKKFCEANKMKSGRRVGVSHKEDGAASFFHTGSKMRKLAFNRIVNYFKGKLSAK

>PdSOFL3

MEASQLAGDAEECSSSESGWTKYLASPMHEGGDSDDHDDVEGDDDDDDDDDDDDDDGGGRSNKNSNGGKDEGDEDDDSMASDASTGRARNKHSCKKGYFPSRRRTEMEKLGILFKGKLVLPLHRAIPRRGSPT

>PdSOFL4

MEASQLAGDAEECSSSESGWTKYLASPMHEGGDSDDHDDVEGDDDDDDDDDDDDDDGGGRSNKNSNGGKDEGDEDDDSMASDASTGRARNKHSCKKGDGIKALEHPKHDEENDGNYSKHSSHLCRIFPKQEKNRDGKTRNPFQREAGSASSQSNPKARKPNLSNK

>PdSOFL5

MESSPLIMEAEECSSCESGWTMYLSSPMHDGGDSDAELQPNDEEEDDANDRCRNESDDGSEGEDNDSMASDASTGPIQRKHGDTKCDQSSLICRLEHDDDDDGIEEDERNQYHLSYSCKKFCEANKMKSGRRVGVSHKEDGAASFFHTGSKVKKASGFNQKKRRHLENDSMNVSLCFCYEKKYRPFYMFSDEKGLQ

>PeSOFL1

MESSQIFVAEECHSSESGWTMYLGSPTQDDDGGDDDEHSEDGDSSDGDGSDNKSYNDDSDDSMASDASSGPSHHGIAHLKQEEDKHVGKYQMEMKANKPKENKKAESGRKEEKEAMAFMEKRANNASAQSGSKVRKTFLMSKRK

>PgSOFL1

MESAQENEDCHSSESGWTMYIGSPAHDDDDDDDDGNTSKSIDEEDEEDEGDDEDGSGNNKNKNGHQEDEETDDSMASDASSGPSYYRELNVPRGGECSSRHKKASQVQEEVSKRSRGDERRRPAKEEADRSETGSKVRKNWKFQMGKRK

>PhSOFL1

MESSSHITGDDGGEGCNSCESGWTMYLASPMHGDDAGGSGKGSGSEGSSVDDGYGYIISDRRSGKKAYEDYADADDDDSLASDASTGPAKEKAPSSLPEDGEKEEDDGGRGKNAGKEEEEEGDVRTKFPTTSRKKAGKVEKGGEGNSSRRGHSKRGSSSRRSFFLW

>PmSOFL1

MDPFRNILSIEGCSSSESGWTTYIASPMQEDDAECSNIEDADYDNHNRITYHKKDKIGKDDNESDDSMASDASSGPSHHHHLVSSRSKDSKAAAGFGSKRDNKESKHKSTRKQEKKSGEKSTKRK

>PmSOFL2

MESSKVFGGAEECHSSESGWTMYIGSCIDGENEDGTGMTHENSEDDGENDGHKDDHSDDSMVSDASSGPSHHHHGGERSRGHGLAVDKTAKKKPKEKQQPRAGRRKEEKKDKALLTHAKKC

>PpSOFL1

MDPFRNIFSIEGCSSSESGWTTYIASPMQEDDAECRNIEDADYDNHNRITYHTKDKIGKDDNESDDSMASDASSGPSHHHHLVSSKSKDSKAAAGFGFKRDNKQSKHKSTRKQEKKSGEKSTKRK

>PpSOFL2

MTYNYPILVLISPASLLIFSPSSLSSKVFGGAEECHSSESGWTMYIGSCTDGENEDGTGMTHENSKDDGENDGHKDDHSDDSMVSDASSGPSHHHHGGERSRGHGLAVDKKAKKKPKEKQQPRAGRRKEEKKDKVLLTHAKKR

>PpSOFL3

MESSQVVGDEPEKWSGSESGWTMYIGSPIHTQTNDVYQISDPKEGGEKNSDKKAILEYFDDDESDDSMASDASSGPISSHHELVLPCEVGEGEGRLRQAAKQDQHGNYFKCSSGKKVGYRKLKKRDERLGIKEEKEEEFLHKADSAGSHV

>PpSOFL4

MESSKVFGGAEECHSSESGWTMYIGSCTDGENEDGTGMTHENSKDDGENDGHKDDHSDDSMVSDASSGPSHHHHGGERSRGHGLAVDKKAKKKPKEKQQPRAGRRKEEKKDKVEVGSIGFS

>PpSOFL5

MGLIVLLCEKLPLLLKGNNKETDINAFPDNVENCTYSDHHVIYTHIAGDEPEKWSGSESGWTMYIGSPIHTQTNDVYQISDPKEGGEKNSDKKAILEYFDDDESDDSMASDASSGPISSHHELVLPCEVGEGEGRLRQAAKQDQHGNYFKCSSGKKVGYRKLKKRDERLGIKEEKEEEFLHKADSAGSHV

>PpSOFL6

MESSKVFGGAEECHSSESGWTMYIGSCTDGENEDGTGMTHENSKDDGENDGHKDDHSDDSMVSDASSGPSHHHHGGERSRGHGLAVDKKAKKKPKEKQQPRAGRRKEEKKDKVLLTHAKKR

>PpSOFL7

MESSKVFGGAEECHSSESGWTMYIGSCTDGENEDGTGMTHENSKDDGENDGHKDDHSDDSMVSDASSGPSHHHHGGERSRGHGLAVDKKAKKKPKEKQQPRAGRRKEEKKDKVLLTHAKKR

>PtSOFL1

MEFSQILGCTEEYSGATGSESGWTKYIASPVKENDFDDDNADSKNKQGDCRKRNYGNDDGGGESDDSMTSDASSGPSHPELPCRSSKGSVNIGPSKYATSKNSSKAKLQKQVKERDGSARIRVENEVSVLKANSAASYVQSGTKVTYMHEDFITSSSWKNEIQGMHPDTVHESGRVPVWLYYILVTDQQGGLADPDD

>PtSOFL2

MDSYKQILGAEGCSSSESGWTTYLASPVQEDEDDEGSYDGNNYKAHNVSNNYHYAAAADEVSDDSMASDASSGPHHQNIHENGRGTVHFKHNKGGHFNLQSSSAKKTGKKDKKCDKNSAKKSRKLDAHRKH

>PtSOFL3

MEFSQTLGREEECSGTSGSESGWTSYIASPIKENNFDDDNDDSKNKQGDCREGNYESDDSMASDASSGPSHPELPCRINEGSINKGPFKNATTKYSSKEKLHRQVKRRDGARTTVKKEVSVLKANSAASHDHVQSGTKMATSKARSMLF

>PtSOFL4

MDSSKHILGSEGCSSSESGWTMYLTSPMQEDDDDQCSYDVNDYNAHDVSKNYRSAADGDSDDSMASDASSGPHHQNTHENGHGVVHFKFSKGGLFNLLSSMAKPEKKDKKSDKNSAKKSRKLDAHRKHK

>PtSOFL5

MESSQIFVAEECHSSESGWTMYLGSPTQDDDGGDDDEHSDDGDSSDGGGSDNKSYNDDSDDSMASDASSGPSHHGIAHLKQEEDKHVGKYQMDQMKANKPKENKKAESGRKEEKEAMAFMEKRANNASAQSGSKVRKTFLMSKRK

>PviSOFL1

MESSQITGDDGEECNSNESGWTMYLGSPVSVDDVKSNETEGSNVESLCSNGRSKPDADYDDGDYDSLASDASTGPAQVKSRNGKEKKDHDKNDSIRDEHGNVEQEEMHTKLPTSCDKKAGKMKKGEDKTTRRSHNKRRSSSRTGFFW

>PviSOFL2

MESSQITGDDGEECNSNESGWTMYLGSPVSIDDVKSNETEGSNVGSVCSNGRSKPDADYDDGDYDSLASDASTGPAQVKIRNGKEKKDHDKNDTIRDEHGNVEQEEMHTKLPTSCDKKPGKMKKGEDKTTRRSHNKRRSSSRTGFFW

>PviSOFL3

MESSSHITGDDGGEGCNSCESGWTMYLASPMHGDDAGGSGKGSGSEGSSVDDGYRYIISDRRSGKKAYEDYADADDDDSLASDASTGPAKEKAPSSLPEDGEKEEDGGRGKNSGAAGKEDEEEGDVGTKFSTTSRKKAGKVDKGGEGNSSRRGNNKRGSSSRRSFFLW

>PviSOFL4

MESSSHITGDDGGEGCNSCESGWTMYLASPMHGDDTGGSGKGSGSEGSSVDDGYGYIISDRRSGKKAYEDYADADDDDSLASDASTGPAKEKAPSSMPEDGEKEEDGGGRGKNGGAGKEDEEEGDARTKFPTTSRKKAGKVDKGGEGNSSRRGHSKRGSSSRRSFFLW

>PvuSOFL1

MEPPHVMLGGEEECHSNESGWTMYIGSPRDEEAHCDDNEEKGYSLDYDYEDAEADPDVESDDSMASDASSGPSQYGVINPLGGGYDGITHIQQQKEEVKEKCCSDQKRITASKSKGNQVVEKRVEKNKMVLMSSKGKAPAV

>PvuSOFL2

MEPFAGEECHSSESGWTMYIGSPMDDGGHSDDGDTDEEGIQTNPQNDDDDDDQSDDSMASDASSGPSHLGINHGFADFHRDAEEEYDADKYCLEKKANKTTQAKQMEGKKVEKKGMLFVDSKDKSPVQGCGKVRNFVGKRK

>RcSOFL1

MESSHVFGGAEECHSSESGWTMYLDSSIHGGNNQLSYNDEEDDHHRYHGGGSGKYYYQEEDSDDSMASDASSGPSLQGMAQFKSEQEKEDYEDDGKYYSTSKLNKQVEKQKKTAQRRRKDEKKEEAGFNAARANSNANNSTPAQSGSKDM

>RcSOFL2

MDSSKHTEGCSSSESGWTMYIASPMQEDGNDCSDDTDDGNHHNDVIINDRHDHADDNEQQDSDDSMASDASSGPHHQYRYENPQRKGPLGNFKHSVGNKFNHCSPAEKTNKKDKKNDGNSNEKNRKLTANRKYSR

>RsSOFL1

MESGKIFGSEEESRSCESGWTMYLASLSNDHGQDCYYDDDGDEDSDGGDSMDSDASSGPMEATANLKLPQEIAEQNSIKKKIKKTNEEMVLVETRVHNNNHDDDDDDDDDNQEFDHDDGNDSYSAIHSYVGPIG

>RsSOFL2

MESGKIFGTEEDSRSCESGWTMYLASYSQDPDDDGYYKDGDDEDSDGGDSMDSDASSGPMEATSTLKLPQEIEEQNSTEKKKKSNEEMVLVEPRVHNNNHYDDDNDDDYDQDDGNDTYSAVHSYVGAVRQGFV

>RsSOFL3

MNSGKIFGSDEDSRSCESGWTTYLVSPHDHDYGNYSDDGDSSGGDSMDSDASSGPVKQIPCLKLPQETTEPNCLKKKKKKANEEKVLVETRVHNDNDDDGDNHDYDDGDNHDYDDGDNHNYDAGNDSHSAVHSYVGSVRQDG

>RsSOFL4

MESPRNHGVSEEEEEYNSCESGWTMYIEDAFHGNDHSYIVADDEDDDDGVDIDDDSKVKEADDGGGDEESDDSMASDASSGPSNQLPKNINKNAARKNVSKQVCIQKSQHAEKTLSNEGEKSELKARTRTSAASRVQSKGKVSKTK

>RsSOFL5

MESPSIHGSAEEKSSCESGWTMYIEDTFHGNHQSEVVYEEHDDDGDGFRVKEVDDEGSSENRSDDSMTSDASSWPSTQLPKNTKNHAVAKRSNATQVYHQTKNRACQKFSDQEEESEFKARTKTTEASRVKSRGKVSKTK

>RsSOFL6

MESPRNHGGPEEEENSSCESGWTMYIGDAFQGDDHSTVVVDDDNDDDSYVKDADDGYENDDDGKESDDSMASDASSGPSNQLPNKNNKHATKQVYIQKRQPTEKTLSNEGEKSEHKARTRTSAASRVQSRGKVSKTK

>RsSOFL7

MESPSIHGSAEEKSSCESGWTMYIEDTFFHGNHQSEVVYEEYDDDGDGFRVKEVDDEGSSENRSDDSMTSDASSWPSTQLPKNTKNHAVAKRSNATQVNHQTKNRACEKFSDQEEESEFKARTKTSEASRVKSRSKVSKTK

>RsSOFL8

MESPTIHGEGAEEKSGCESGWTMYIEDTFHGNHPSEVVYEEYDDDFRFKKVDDEDAEDGDGDDSSENGSDDSMTSDASSWPSTQHQRNIKNHAAAKKSNAKQVSHKTKNRACEKFSDEEEESEFKARTRTTTTTRVQSRGKVSKTK

>SbSOFL1

MESSSHITGEDDGEGCNSCESGWTMYLASPMHMHGHAGDDAAGSGKSSGSQGSSVDDGYGYMISRGNQQGYQHYADADDDDSLASDASTGPAKVKSSPSSSPPEYQQRRKDDDDDDGGHHHGHGKEQEEDDGARTKLATSSRKKAAAGNGGGGGAEGNSSRRGHSKRGGGSSRRSFFLW

>SiSOFL1

MESSQINGDGGEECNSNESGWTMYLGSPVDSDDAETNGSEGSNVSSGRSNGRSKNTDADYDSLASDASTGPAQVKMRDGKEKKSQDKNDSIRYEHGNDRQEEIHTKLTISCDKKAGKMNKREEKTARRGHNKRRSSSRTGLF

>SiSOFL2

MESSSHITGDDGGEGCNSCESGWTMYLASPMHGDDAGGSGKGSGSEGSSVDDGYGYIISDRRSGKKAYEDYADADDDDSLASDASTGPAKVKSPSSPPEDGRKEDGGHGKNGVAGKEDEEEEGDVRTKFPSTSRKKGGKVDKGGEGNSSRRGNSKRGSSSRRSFFLW

>SlSOFL1

MDPKSHIFNEGEECHSSESGWTMYIGSPTNDEDDEMNCEGDFDDEDGVLGQGRRKKIMNVVVDDDDDDDDTDDSMASDASSGPSHHIVRNANKSIIIPKEKGNGNNKASLKKGSGKNQDKGRYSVFSAKVPSSSEKVKKSIWKGKGK

>SlSOFL2

MDSNSQFFGEECNSSESGWTMYIGSPSTGDENIGDFDELEESNYNKEGRVNEDDEDCETDDSMASDASSGPISNFSRNAKSGGVDTMVNFKNQKEKGKEKNCSFISNKAAKSSMNNGYKNGDQDKVKEPIVAAKGIKGASNDGGNKVRKTICMGKGK

>SlSOFL3

MEYPKHPGGIEECNSNESGWTMYIGSPYREYNDEDDDEGTPMKGDDHVEDGGSDDSMTSDASSGPSHQGVVLCTNIEQIYGKHVEKDNRKFSSKEQLQQQKQAKKKLSDKNTKAAKEDSGHKAKSGKGYGYCRSTTRGKHVS

>SpSOFL1

MEYPKHPGGTEECNSNESGWTMYIGSPYREYNDEDDDEGTPMKGDDHVEDGGSDDSMTSDASSGPSHQGVVLCTNVEQIYGKHVEKDNRKFSSKEQLQQQKQAKKKLSDKNTKAAKEDSGHKAKSGKGYGYCRSTTRGKHVS

>StSOFL1

MEYPKHPGGTEECNSNESGWTMYIGSPYCEYNQIDDDDDEGTPMKGDDHVEDGGSDDSMTSDASSGPSHQGVVLCTNMEQIYGKHVEKDIRKFSSKEQLQQQKQAKKKLSDKNTKAAKEDSGHKAKSGKGYGYCRSTTRGKHVS

>StSOFL2

MDSNSQFFGEGEECNSSESGWTMYIGSPSTGDDNIGDFDELEDGNYNKEGPVNEDDEDCETDDSMASDASSGPISNFSRNGKSGGVATMVNFKNSKEKGKEKNGSFISNKAKSSMKNGYKNDDQDKVKESIIAAKGTKGASNGGGNKVRKSIWMGKGK

>StSOFL3

MEYPKHPGGTEECNSNESGWTMYIGSPYCEYNQIDDDDDEGTPMKGDDHVEDGGSDDSMTSDASSGPSHQGVVLCTNMEQIYGKHVEKDIRKFSSKEQLQQQKQAKKKLSDKNTKAAKEDSGHKAKSGKGYGYCRSTTRGKHVSQGVAAAEKKGKKKPAKEKQTRGGRRKQEEKKEKALLIHGKKR

>StSOFL4

MDPKSHIFNEGEECHSSESGWTMYIGSPTNDEDDEMNCEGDFDDEDGVLGQGRRKKIINDDDDTDDSMASDASSGPSHHIVRNANKSTIIPKEKGNGKNGSNNKASMKKGSGKNQDKGGYSVFSAKVPSSSEKVKKSIWKGKGK

>SvSOFL1

MESSSHITGDDGGEGCNSCESGWTMYLASPMHGDDAGGSGKGSGSEGSSVDDGYGYIISDRRSGKKAYEDYADADDDDSLASDASTGPAKVKSPSSPPEDGRKEDGGHGKNGVAGKEDEEEEGDVRTKFPSTSRKKGGKVDKGGEGNSSRRGNSKRGSSSRRSFFLW

>TcSOFL1

MDPLKHFPSKEGCSSSESGWTRYLASPMQDDFECSEDNYNKYSIKDDDDNDGDDGEGNSDDSMVSDASSAPSHHQYKHKDGQGSHSSSHLKHDEGDYAGKHSSRKEGKKEAKKSVENSGKSKRRLGGQAKSGK

>TcSOFL2

MEPSKILGGTEECHSSESGWTMYIGSPIQGGDDDDDDGHSDADAYAANYGGHADETEINHEADSDDSMASDASSGPSHQGHRYGNMEEGHGTSHLNDEVEGEGNYYLDKEAKKSLEKQKLGMKKKEDKEDQKERMTLKAKGAATPRSGSKVRKSIWLGKRK

>TcSOFL3

MEASHILGGMEDEKHSSSESGWTMYIGSSTRENDRYNYIDEYDCDTHEQEEDNHKNHRGNYDGNSHNDDESDDSMASDASSGPSHHKLPCSSEQNLGMDRHKHEMLKSTSTEKLHKQVIKRDQRRNKIEKEKLERKAISAASHVRGGEKVKTINIMSQEE

>ThSOFL1

MEPEKAIGSEEECRSCESGWTMYLVSPSHDHHDDHGHDGDAHHREDDSDGDSMASDASSGPVEATCRLSRTEEGLNCNYGLCLTKRTGKPVMKKTTTNTKEGKEERVLTETRVPTIAHDDDDDSSEVHSYVGSI

>ThSOFL2

MESAKIFGSEEECRSCESGWTMYLVSPSHGHDDYYYGGDDDHGHGNDGSANQHQHHEEDHSDVDSMASDASSGPMEASLQLLNRTQERNCSNYGLCLTKKTGKPVIKNKNKKKKKKNMKTKEEERTVSTESRVPKIVHNNNNDGDDDDDDDDDDDGDDSSDVHSCVGSVKPDGYV

>ThSOFL3

MVDEENSSGESGWTMYLDDAFNGNEHSGVVDDFHERDDDDDDDDDDDSMASDASSGPSNQLQRDNTKHAAAENSSKQQVIIHQHHQLTEKAKKKSSADEKKKSLLNARTRTTAASHVQTRNKMSKTT

>ThSOFL4

MEKEECRSNESGWTMYISSPMEEDDEEVVDENYYDEGHYVYRECRKGHKEDENNKDSDDSMASDASSGPSYQHYNQKRNSETHPKINRGRRREGFVSRSGKGDSVGKCYDEDDSYGRNDNSSNYKGKKKEKKNGENNKTRHRMK

>TsSOFL1

MDSFKHIWGAPEGCSSSESGWTMYIDSPMQEDDARCSNENDGYNHNHHEIYGEKTRRKKKVDEEESDDSMASDASSGPTNYQSYARGGQGSQGRSVSKKDHKQDHGSKSCSKKNGNKQEKKRVDSRSKK

>TsSOFL2

MEQPLHMLLGAEEECHSSESGWTMYIGSPIKDGNLDDDDDDDEVDFYQGIHQTKGDAGIESDDSMVSDASSGPSHYGVNVIHPLGNYEGGYGLRHFMQKVDEYDDDDDELHDYYCFDHHKKGSKKKENQIGEKKGEKKQKVQVQGGGRFKGRKNQRMGTRK

>TuSOFL1

MESSHITGDGGEECNSNESGWTIYLTSPTSSYEAKENGSEGSNVEDGSGYITERRKGKEENNADDDGDYDSLASDASTGTSQVKVLEGKEEKDRQTNDDCSNEHGKDEQAETLTKFSTGSNKKAGKAARGYDATGLEWWRKAAEQSTVVTTAKRPSANISDIPATARSSISCAPWNTAPSDILFPSLPRPLASPRLRRALLGGSPWLYHGSGEAGRARGGGGGEAAGHAARRGQKGRRRADHLDERSCHL

>VaSOFL1

MDSFKQIWGSAEGCSSSESGWTMYIASPMQEDDAGCSNENYGNHDIYGDNRRKKQGAKVDEEESDDSMASDASSGPVHYHHAYGHSQTSHGTAASKKDKQDHGSKCSKKNASKQEKKRVDSRSKK

>VaSOFL2

MEPPHLRMGGEEECHSNESGWTMYIGSPRDEDAHCDDNEDKGFSLDYDYEEAEADADVESDDSMASDASSGPSQYGVSNPLGGGCDGITQFQQKEEGKNKYSSDHKAKGNHQVVEKRAEKNHKKMVLISSKGKAPAV

>VrSOFL1

MDSFKQIWGSAEGCSSSESGWTMYIASPMQEDDAGCSNENYGNHDIYGENRRKKQGAKVDEEESDDSMASDASSGPVHYHHAYGHSQTSHGTAASKKDKQDHGSKCSKKNASKQEKKRVDSRSKK

>VrSOFL2

MEPPHVRMGGEEECHSNESGWTMYIGSPRDEDAHCDDKEEKGFSFDYDYEEAEADADVESDDSMASDASSGPSQYGVSNPLGGGCDGITQFQQKEEVKNKYCSDHKASKSKGNHQVVEKRAEKNHKKMVLISSKGKAPAV

>VvSOFL1

MEPKHFPGTEGCSSSESGWTMYIASPMHEGDSECSGNDDDNNIHITNFNYGDGKDEGSDDSMASDASSGPSHHEQACENGQDSHGMGMAPFQHDRGDNIXKYSSWXKGNKQEKKKGGESSSKKEKKDLVLRRNSASSHTHRGAIAIIYSKQSVSAINHKFQVSNQNALFDKXEFKESTWKXVKMGIKSNEELXHQI

>VvSOFL2

MEPKHFPGTEGCSSSESGWTMYIASPMHEGDSECSGNDDDNNIHITNFNYGDGKDEGSDDSMASDASSGPSHHEQACENGQDSHGMGMAPFQHDRGDNIKKYSSWEKGNKQEKKKGGESSSKKEKKDLVLRRNSASSHTHRGASK

>ZjSOFL1

MGTEECSSSESGWTMYIASPVEEDDAECSSCGDDNDAKSKKRVTYQTYAANEEDFGSDDSMASDASSGPSYQQYYHGRSHCKESHGKKDKKQSTNKNNTNKKEKKSSGEKNTKRK

>ZjSOFL2

MMDSSQSFGGAEECQSSESGWTMYIGSPIHDDDQYGSDYADNDDGGTNDGDDDDNSDADHHNDENDSDDSMASDASSGPSHLGRTNGNAVFKDEEEENDGKYCIIDEKAKKPSMEKMQRSAERRKEEMVFMGRKAKAPVQNGAKKVQKTIWTGKRK

>ZmSOFL1

MDSSQLTGDNAEECNSNESGWTMYLGSPVNSDDLKANGSKWSNVGSGCSNGRSKNDNMDYDDGDYDSLASDASTGPAQVKVLNGKEKKIHEKRDNSIEEQDSDEQDEQTKLPNNCDKKAGKMKKEEKTTRRSHNKRRSSCSSRTSFLR

>ZmSOFL2

MRRESKATWTTGGNTWNTALQANVLPAAFGSNRRSRSENKKAFFEVGLLRCSEELTGDNAEECNSNESGWTMYLGSPVNSDDLKANGSKWSNVGSGCSNGRSKNDNMDYDDGDYDSLASDASTGPAQVKVLNGKEKKIHEKRDNSIEEQDSDEQDEQTKLPNNCDKKAGKMKKEEKTTRRSHNKRRSSCSSRTSFFR

>ZmSOFL3

MESFSHITGGDDDAGGEGCNSCESGWTMYLASPMHMHGHDDDDDAGGSANQGSSVDDGYGYMINGGGNKKQDYDDDDGDGDSLASDASTGPAKAANKSPSPPPEHKHGKEDDDRGHRGGGGGGKEETKLATSSRKKAAAGNGKLMDKAAAAAAGGGEGNSSRRGHGKGGGGGSSRRSFFLW

>ZmSOFL4

MESSSHITGGDDDAGGEGCNSCESGWTMYLASPMHMHGHDDDDDAGGSANQGSSVDDGYGYMISGGGNKKQDYDDDDGDGDSLASDASTGPAKAANKSPSPPPEHKHGKEDDDRGHRGGGGGGKEETKLATSSRKKAAAAGNGKLMDKAAAAAAGGGEGNSSRRGHGKGGGGGSSRRSFFLW

**File S1. SOFL orthologues protein sequences.** 289 SOFL orthologous protein sequences were extracted from NCBI (Sayers et al., 2009) and Phytozome (Goodstein et al., 2011) databases after searches using the three founding SOFL members as queries.
