# Supplementary material for: Synopsis of the SOFL Plant-Specific Gene Family
Source: G3 (Bethesda). 2018 Feb 23;8(4):1281–90. doi: 10.1534/g3.118.200040 (PMC5873917; doi:10.1534/g3.118.200040)
Supplement: Supplementary file 2 [file 1281FileS2.docx]

**Protein sequence retrieved from our NCBI database search using founding *Arabidopsis thaliana* *SOFL* members as queries**

(A) GenBank_AAG50669.1

MESPRNHGGSEEEEYSSCESGWTMYIEDAFHGNDQSSVVVDDDDDDTQVKEADDGYENDDGDTSDDGGDEESDDSMASDASSGPSNQLPKHINKHAARKNGSKQVYLQKRQHTEKTISNEGEKSDLKARTRTSAASRVQSRGKDKVVDDKTGVSKVANMVSMMCGPSS

(B) GenBank_AC079829.6

ATGGAGTCTCCGAGAAACCATGGAGGCTCTGAAGAGGAGGAATACAGCAGTTGTGAGTCAGGGTGGACTATGTACATAGAAGACGCCTTCCATGGAAATGACCAATCATCTGTTGTTGTCGATGATGATGATGATGATACACAGGTTAAAGAGGCTGATGATGGTTATGAGAACGATGATGGCGATACTAGTGATGATGGTGGTGATGAAGAGAGTGATGATTCCATGGCTTCTGATGCATCCTCGGGGCCTAGCAATCAGCTTCCAAAGCACATCAACAAACATGCAGCTAGGAAGAATGGTTCTAAACAAGTCTACCTTCAAAAACGCCAACACACAGAGAAAACAATCAGCAACGAAGGAGAAAAATCAGACCTCAAAGCTAGAACAAGAACAAGTGCAGCTAGTCGTGTCCAGAGTAGAGGGAAGGACAAAGTGGTTGATGACAAAACAGGAGTTTCTAAGGTTGCCAATATGGTGTCTATGATGTGTGGCCCAAGTAGCTAA

**Sequencing results of gDNA and cDNA PCR products**

(C) Sequenced_At1g26210_genomic_sequence

**ATGGAGTCTCCGAGAAACCATGGAGGCTCTGAAGAGGAGGAATACAGCAGTTGTGAGTCAGGGTGGACTATGTACATAGAAGACGCCTTCCATGGAAATGACCAATCATCTGTTGTTGTCGATGATGATGATGATGATACACAGGTTAAAGAGGCTGATGATGGTTATGAGAACGATGATGGCGATACTAGTGATGATGGTGGTGATGAAGAGAGTGATGATTCCATGGCTTCTGATGCATCCTCGGGGCCTAGCAATCAGCTTCCAAAGCACATCAACAAACATGCAGCTAGGAAGAATGGTTCTAAACAAGTCTACCTTCAAAAACGCCAACACACAGAGAAAACAATCAGCAACGAAGGAGAAAAATCAGACCTCAAAGCTAGAACAAGAACAAGTGCAGCTAGTCGTGTCCAGAGTAGAGGGAAGGTGAGCAAAACCAAATAA**AACTCATGGCGAATGAGAGAAGAAGATATGGGGGTTCACAAAGAATCCAAATCCTGTAGTGAAGAGAGACGTTTCAGGGGTCTCACC**AATCGTGTTGTTTGCCTTTAATAAGCCATGTGTAGGTAG***TTGCATTGTTTTCTTTTGCCGGAATATGTTTTTTTGTAGAAGCTGTCCCAAATAAGTTATCTGCACGGTTCTCCGTTGGAACCCTGTTATATAATATGTACCTTGTTCTTCAGAGACTTCCTTCTAACACAAAACAGAGCATATCCTGATCTGCATGTTCAACACAGGAGCCATCAAGATTCAAGAGAGTGATTCTTGGAAGAAAAACCAACAGTTCCTGAATCATTCTCAACAGCAACCAGATAATAACCAATCTTTAGCAATTGAAAAGCAAAACATTAGTTATAAAATACTAATAATCTTTTTCCGAGAAAAAAGAAACGCTCATGGTAGAGGACAAAGTGGTTGATGACAAAACAGGAGTTTCTAAGGTTGCCAATATGGTGTCTATGATGTGTGGCCCAAGTAGCTAA*

(D) Sequenced_At1g26210.2_CDS_sequence

**ATGGAGTCTCCGAGAAACCATGGAGGCTCTGAAGAGGAGGAATACAGCAGTTGTGAGTCAGGGTGGACTATGTACATAGAAGACGCCTTCCATGGAAATGACCAATCATCTGTTGTTGTCGATGATGATGATGATGATACACAGGTTAAAGAGGCTGATGATGGTTATGAGAACGATGATGGCGATACTAGTGATGATGGTGGTGATGAAGAGAGTGATGATTCCATGGCTTCTGATGCATCCTCGGGGCCTAGCAATCAGCTTCCAAAGCACATCAACAAACATGCAGCTAGGAAGAATGGTTCTAAACAAGTCTACCAATCGTGTTGTTTGCCTTTAATAAGCCATGTGTAG***GTAGTTGCATTGTTTTCTTTTGCCGGAATATGTTTTTTTGTAGAAGCTGTCCCAAATAAGTTATCTGCACGGTTCTCCGTTGGAACCCTGTTATATAATATGTACCTTGTTCTTCAGAGACTTCCTTCTAACACAAAACAGAGCATATCCTGATCTGCATGTTCAACACAGGAGCCATCAAGATTCAAGAGAGTGATTCTTGGAAGAAAAACCAACAGTTCCTGAATCATTCTCAACAGCAACCAGATAATAACCAATCTTTAGCAATTGAAAAGCAAAACATTAGTTATAAAATACTAATAATCTTTTTCCGAGAAAAAAGAAACGCTCATGGTAGAGGACAAAGTGGTTGATGACAAAACAGGAGTTTCTAAGGTTGCCAATATGGTGTCTATGATGTGTGGCCCAAGTAGCTAA*

**Comparing *AtSOFL1.1* and *AtSOFL1.2* coding sequences and genomic sequences, respectively.**

(E) AT1G26210_AtSOFL1.1_CDS

**ATGGAGTCTCCGAGAAACCATGGAGGCTCTGAAGAGGAGGAATACAGCAGTTGTGAGTCAGGGTGGACTATGTACATAGAAGACGCCTTCCATGGAAATGACCAATCATCTGTTGTTGTCGATGATGATGATGATGATACACAGGTTAAAGAGGCTGATGATGGTTATGAGAACGATGATGGCGATACTAGTGATGATGGTGGTGATGAAGAGAGTGATGATTCCATGGCTTCTGATGCATCCTCGGGGCCTAGCAATCAGCTTCCAAAGCACATCAACAAACATGCAGCTAGGAAGAATGGTTCTAAACAAGTCTACCTTCAAAAACGCCAACACACAGAGAAAACAATCAGCAACGAAGGAGAAAAATCAGACCTCAAAGCTAGAACAAGAACAAGTGCAGCTAGTCGTGTCCAGAGTAGAGGGAAGGTGAGCAAAACCAAATAA**

(F) Sequenced_Putative_AtSOFL1_genomic_sequence **ATGGAGTCTCCGAGAAACCATGGAGGCTCTGAAGAGGAGGAATACAGCAGTTGTGAGTCAGGGTGGACTATGTACATAGAAGACGCCTTCCATGGAAATGACCAATCATCTGTTGTTGTCGATGATGATGATGATGATACACAGGTTAAAGAGGCTGATGATGGTTATGAGAACGATGATGGCGATACTAGTGATGATGGTGGTGATGAAGAGAGTGATGATTCCATGGCTTCTGATGCATCCTCGGGGCCTAGCAATCAGCTTCCAAAGCACATCAACAAACATGCAGCTAGGAAGAATGGTTCTAAACAAGTCTACCTTCAAAAACGCCAACACACAGAGAAAACAATCAGCAACGAAGGAGAAAAATCAGACCTCAAAGCTAGAACAAGAACAAGTGCAGCTAGTCGTGTCCAGAGTAGAGGGAAGGTGAGCAAAACCAAATAA**AACTCATGGCGAATGAGAGAAGAAGATATGGGGGTTCACAAAGAATCCAAATCCTGTAGTGAAGAGAGACGTTTCAGGGGTCTCACCAATCGTGTTGTTTGCCTTTAATAAGCCATGTGTAGGTAGTTGCATTGTTTTCTTTTGCCGGAATATGTTTTTTTGTAGAAGCTGTCCCAAATAAGTTATCTGCACGGTTCTCCGTTGGAACCCTGTTATATAATATGTACCTTGTTCTTCAGAGACTTCCTTCTAACACAAAACAGAGCATATCCTGATCTGCATGTTCAACACAGGAGCCATCAAGATTCAAGAGAGTGATTCTTGGAAGAAAAACCAACAGTTCCTGAATCATTCTCAACAGCAACCAGATAATAACCAATCTTTAGCAATTGAAAAGCAAAACATTAGTTATAAAATACTAATAATCTTTTTCCGAGAAAAAAGAAACGCTCATGGTAGAGGACAAAGTGGTTGATGACAAAACAGGAGTTTCTAAGGTTGCCAATATGGTGTCTATGATGTGTGGCCCAAGTAGCTAA

**Translated protein sequence from putative *AtSOFL1.2* CDS sequence**

(G) Translated_Putative_AtSOFL1.2_protein_sequence

MESPRNHGGSEEEEYSSCESGWTMYIEDAFHGNDQSSVVVDDDDDDTQVKEADDGYENDDGDTSDDGGDEESDDSMASDASSGPSNQLPKHINKHAARKNGSKQVYQSCCLPLISHV*VVALFSFAGICFFVEAVPNKLSARFSVGTLLYNMYLVLQRLPSNTKQSIS*SACSTQEPSRFKRVILGRKTNSS*IILNSNQIITNL*QLKSKTLVIKY**SFSEKKETLMVEDKVVDDKTGVSKVANMVSMMCGPSS*

**File S2. Annotation of *AtSOFL1* and the two alternative splicing variants.**
