# Supplementary figures and images for: Synopsis of the SOFL Plant-Specific Gene Family
Source: G3 (Bethesda). 2018 Feb 23;8(4):1281–90. doi: 10.1534/g3.118.200040 (PMC5873917; doi:10.1534/g3.118.200040)

## Slide 1
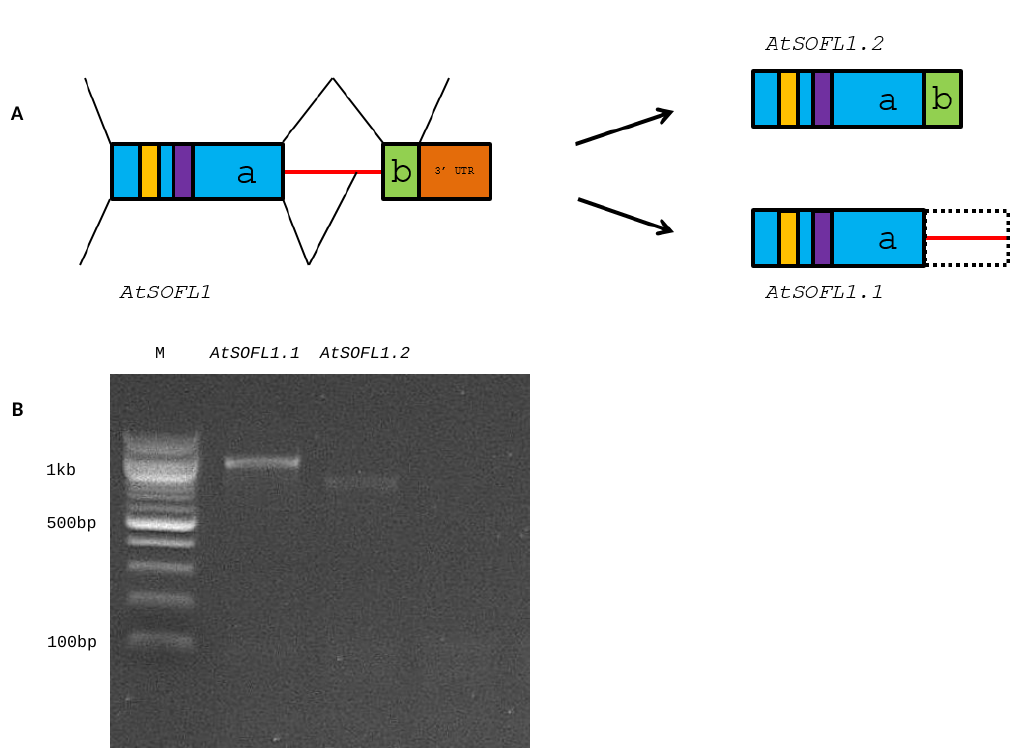

A
 M AtSOFL1.1 AtSOFL1.2
 B
1kb
500bp
100bp

Supplement: Supplementary file 3 [file 1281FigureS1.pptx]

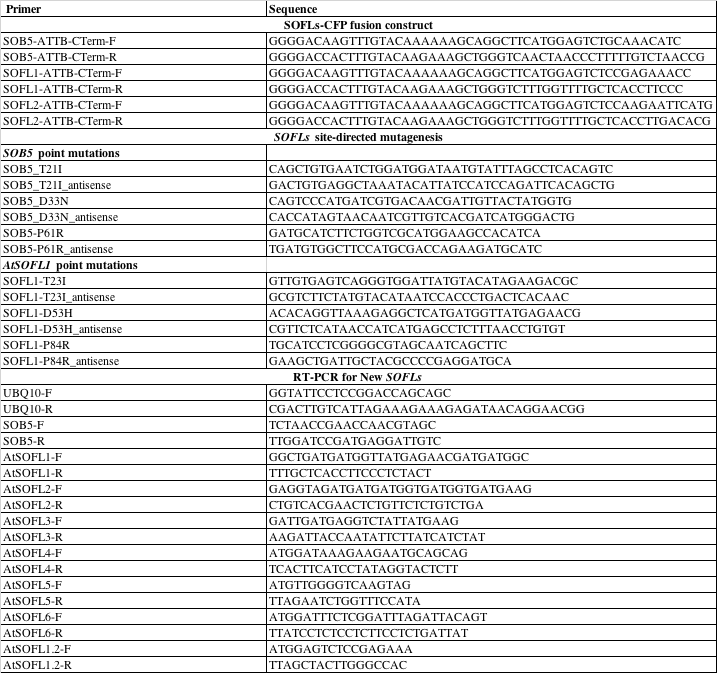

Supplement: Supplementary file 4 [file 1281TableS1.docx]

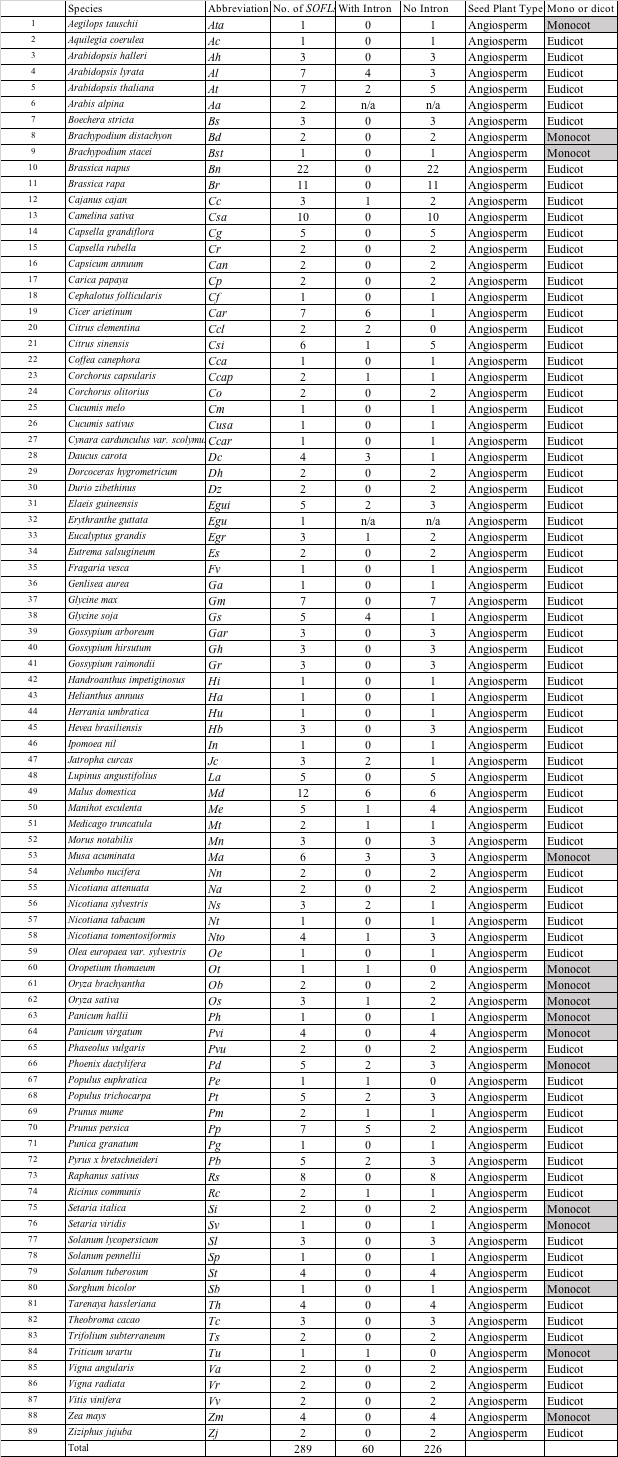

Supplement: Supplementary file 5 [file 1281TableS2.docx]

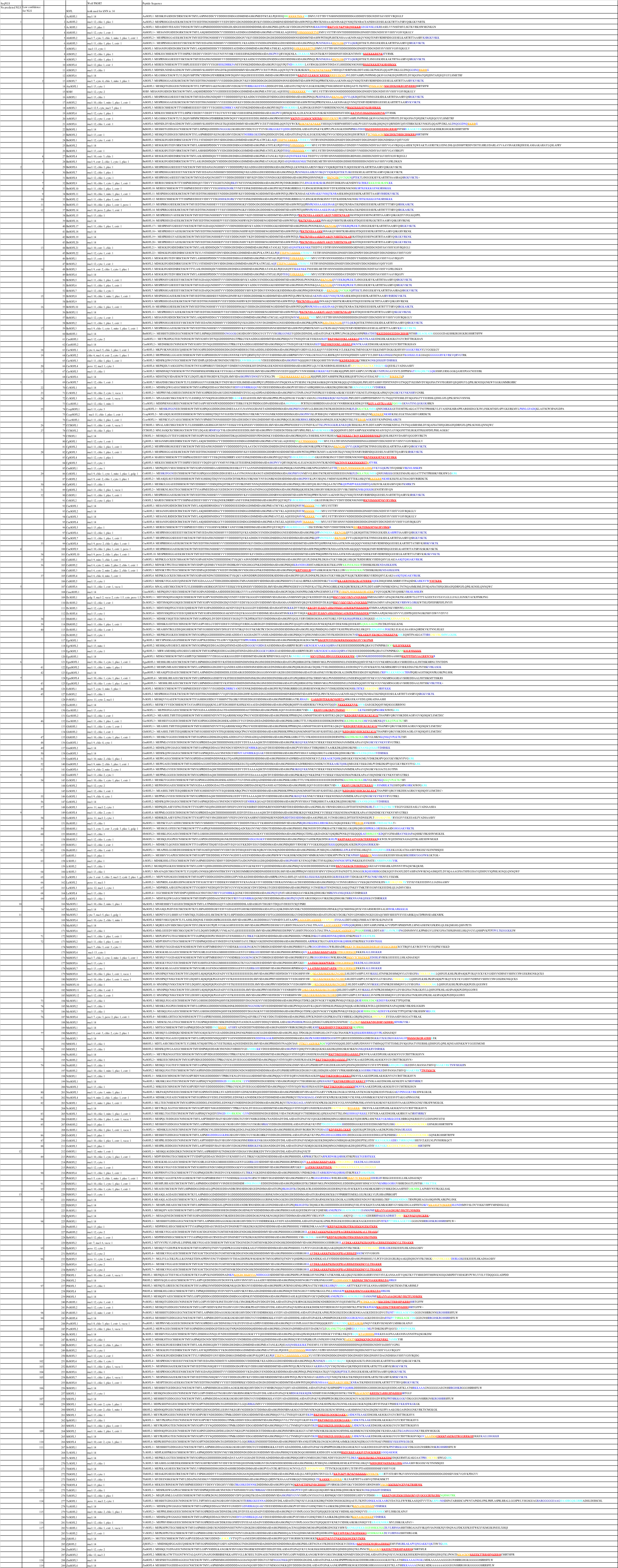

Supplement: Supplementary file 6 [file 1281TableS3.docx]
